# Hydrogel Electrolytes for Zinc-Ion Batteries: Materials Design, Functional Strategies, and Future Perspectives

**DOI:** 10.1007/s40820-025-01993-w

**Published:** 2026-01-05

**Authors:** Zhengchu Zhang, Yongbiao Mu, Lijuan Xiao, Hengyuan Hu, Tao Xue, Limin Zang, Eiichi Sakai, Meisheng Han, Chao Yang, Lin Zeng, Jianhui Qiu

**Affiliations:** 1 Present Address: Department of Machine Intelligence and Systems Engineering, Faculty of Systems Science and Technology, Akita Prefectural University, Yurihonjo, Japan; 2 Shenzhen Key Laboratory of Advanced Energy Storage, Department of Mechanical and Energy Engineering, Southern University of Science and Technology, Shenzhen, 518055 People’s Republic of China; 3 MOE Key Laboratory of New Processing Technology for Nonferrous Metal and Materials, Guangxi Key Laboratory of Optical and Electronic Materials and Devices, Key Laboratory of Natural and Biomedical Polymer Materials (Education Department of Guangxi Zhuang Autonomous Region), College of Materials Science and Engineering, Guilin University of Technology, Guilin, 541004 People’s Republic of China; 4 Present Address: SUSTech Energy Institute for Carbon Neutrality, Southern University of Science and Technology, Shenzhen, 518055 People’s Republic of China

**Keywords:** Zinc-ion batteries, Hydrogel electrolytes, Dendrite growth, Functional optimization strategy

## Abstract

With the escalating demand for safe, sustainable, and high-performance energy storage systems, hydrogel electrolytes have emerged as promising alternatives to conventional liquid electrolytes in zinc-ion batteries. By integrating the high ionic conductivity of liquid electrolytes with the mechanical robustness of solid frameworks, hydrogel electrolytes offer distinct advantages in suppressing zinc dendrite formation, enhancing interfacial stability, and enabling reliable operation under extreme environmental conditions. This review systematically summarizes the fundamental characteristics and design criteria of hydrogel electrolytes, including mechanical flexibility, ionic transport capabilities, and environmental adaptability. It further explores various compositional design strategies involving natural polymers, synthetic polymers, and composite systems, as well as the incorporation of electrolyte salts and functional additives. In addition, recent advances in functional optimization, such as anti-freezing properties, self-healing abilities, thermal responsiveness, and biocompatibility, are comprehensively discussed. Finally, the review outlines the current challenges and proposes potential directions for future research.

## Highlights

- Provides a comprehensive overview of the fundamental properties and structural components of hydrogel electrolytes, systematically summarizing key material elements and performance tuning strategies.
- Focuses on the functional characteristics of hydrogel electrolytes, outlining mechanisms for enhanced performance and adaptability across diverse application scenarios.
- Analyzes the core challenges currently facing hydrogel electrolytes and proposes future development pathways centered on green, safe, and multifunctional integrated optimization.

## Graphical abstract

**Figure:**
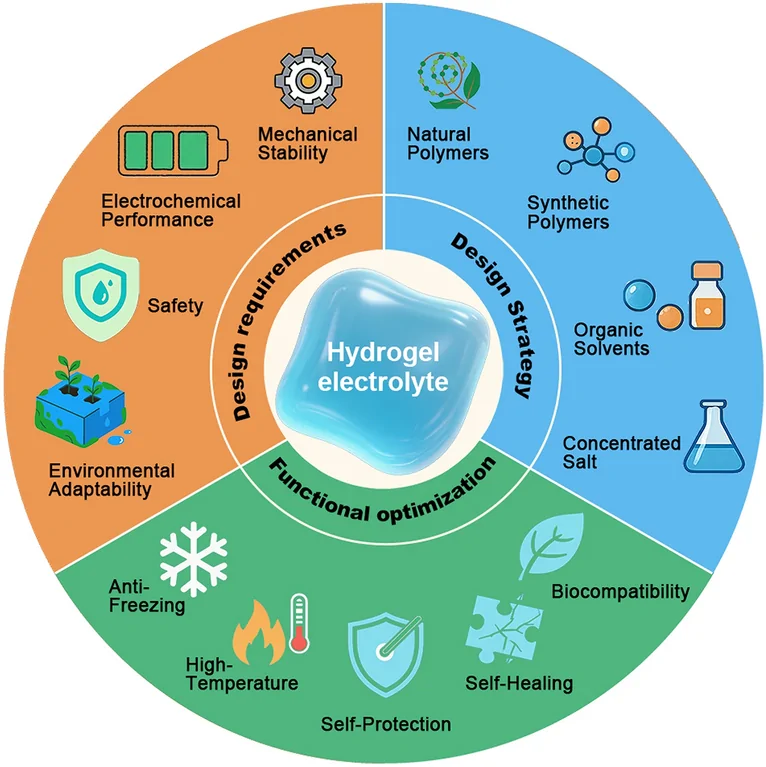

## Introduction

Amid the global transition toward cleaner and more sustainable energy systems, the development of safe, efficient, and environmentally friendly energy storage technologies has become essential for the large-scale integration of renewable energy sources [1–3]. According to the International Energy Agency’s Net Zero by 2050 roadmap, achieving net-zero global carbon emissions by 2050 will require global energy storage capacity to increase to approximately 780 GW by 2030, far surpassing current levels [4]. This soaring demand is accelerating advancements in energy storage technologies, with electrochemical energy storage systems gaining significant attention due to their fast response, high energy density, and wide applicability in smart grids, wearable electronics, and portable devices [5, 6].

At present, lithium-ion batteries, as the most commercially mature electrochemical energy storage technology, have been widely deployed. However, issues such as the risk of thermal runaway, limited lithium resources, and associated environmental concerns hinder their scalability for large-scale applications [7, 8]. In contrast, zinc-ion batteries (ZIBs), which utilize aqueous electrolytes, offer several intrinsic advantages, including high safety, low cost, environmental friendliness, high theoretical capacity, and the natural abundance of zinc [9, 10]. These attributes position ZIBs as promising candidates for next-generation green energy storage systems.

Despite these advantages, conventional liquid electrolytes in ZIBs face several critical challenges [11, 12]. Firstly, the uncontrolled deposition of zinc ions in traditional aqueous electrolytes often leads to dendrite formation, significantly boosting the risk of internal short circuits [13, 14]. Secondly, the fluidity of liquid electrolytes raises the potential for leakage, especially under mechanical deformation or extreme temperatures, which can result in device failure. More critically, unfavorable parasitic reactions like hydrogen and oxygen evolution may occur during operation, jeopardizing both the safety and the cycling stability of ZIBs [15, 16].

To overcome these limitations, namely dendrite growth, electrolyte leakage, and parasitic reactions, research efforts have increasingly focused on gel-state electrolyte systems [17, 18]. In this context, hydrogel electrolytes represent a unique intermediate class of electrolytes that combine the high ionic conductivity of liquid systems with the mechanical stability and shape stability of solids. Unlike purely liquid electrolytes, hydrogel electrolytes suppress leakage and parasitic side reactions, while their flexibility and processability distinguish them from rigid solid electrolytes, features particularly valuable for flexible and wearable devices. Acting as hybrid electrolytes, they offer effective suppression of dendrite formation and mitigate side reactions, such as leakage and gas evolution [19–21].

**Fig. 1:**
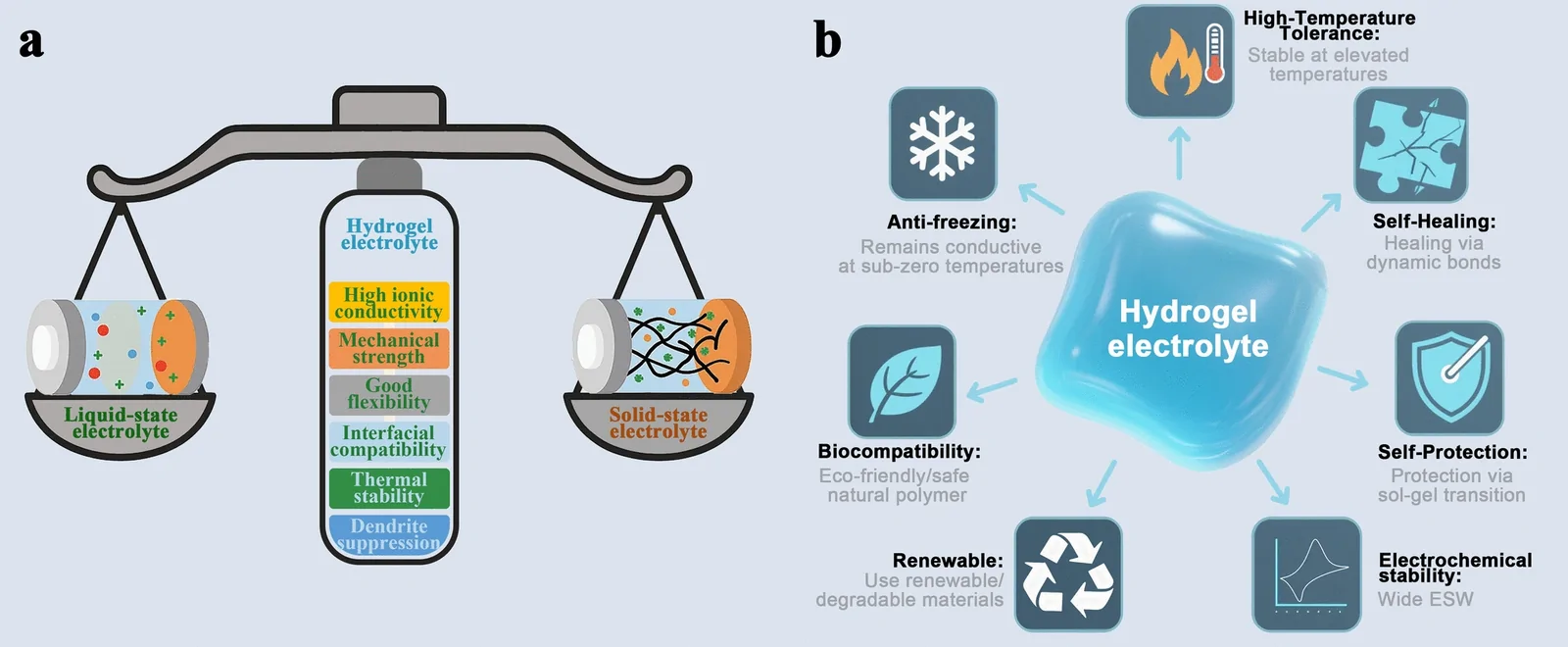
**a** Schematic illustration of the performance balance achieved by hydrogel electrolytes between liquid and solid electrolytes. **b** Schematic illustration of functional properties enabled by hydrogel electrolyte design

Furthermore, the incorporation of renewable resources, green solvents, and functional polymers into hydrogel systems aligns with broader sustainability goals, enabling the development of environmentally compatible and multifunctional electrolytes. Through material design and functional modification, hydrogel electrolytes can be engineered to exhibit excellent environmental adaptability, including anti-freezing capability at low temperatures, dehydration resistance under heat, and properties such as self-healing and self-protection (Fig. 1b) [22–24]. These multifunctional characteristics make hydrogel electrolytes highly suitable for a wide range of demanding energy storage scenarios, presenting them as viable candidates to replace traditional liquid electrolytes [25–27].

Nevertheless, enhancing the performance of hydrogel electrolytes for application-specific environments remains a major challenge in material design. This directly influences the stability, safety, and longevity of devices under harsh conditions, thereby affecting their practical deployment [28, 29]. Consequently, the synergistic optimization of functional properties and electrochemical performance has become a focal point of research and a critical frontier in the development of hydrogel electrolytes. Such efforts not only advance the bulk properties of these materials but also lay the foundation for their real-world application in ZIBs, warranting thorough investigation and comprehensive review. In this context, this present review highlights recent advances in the research and application of hydrogel electrolytes for ZIBs. It begins by discussing their fundamental characteristics and design requirements. Subsequently, it systematically examines construction strategies from the perspective of composition, including the selection and tuning of polymer matrices, electrolyte salts, and functional additives. Finally, it focuses on recent developments in functional optimization, encompassing properties such as wide temperature adaptability, self-healing, flame retardancy, anti-aging, biocompatibility, and sustainability. By critically reviewing current achievements and unresolved challenges, this work aims to provide theoretical insights and developmental guidance for future innovations and applications of hydrogel electrolytes.

## Basic Characteristics and Design Requirements of Hydrogel Electrolytes

As a type of dual-phase composite material, hydrogel typically consists of a three-dimensional (3D) porous network structure formed by hydrophilic polymers through physical or chemical cross-linking, within which a large quantity of water is encapsulated [30]. This unique combination of solid polymeric networks and liquid water endows hydrogels with a suite of distinctive properties, including liquid-like ionic conductivity, solid-like mechanical stability, high specific surface area, optical transparency, and excellent biocompatibility [31, 32]. Owing to these multifunctional attributes, hydrogels have been extensively studied and applied across a range of fields, including energy storage, sensing, bioengineering, and soft robotics [33].

In the context of ZIBs, hydrogels serve as promising electrolyte materials, offering enhanced electrochemical stability while mitigating issues commonly associated with conventional liquid electrolytes, such as leakage and corrosion. To comprehensively assess the advantages and application potential of hydrogel electrolytes in ZIBs, it is crucial to first examine their fundamental characteristics. This includes an evaluation of their intrinsic physical and chemical properties, as well as the key performance indicators relevant to practical energy storage applications.

### Basic Characteristics

This subsection focuses on the intrinsic physicochemical characteristics that define the fundamental behavior of hydrogel electrolytes, laying the groundwork for understanding how their internal structure and composition govern ion transport, mechanical performance, and interfacial stability. For hydrogel electrolytes to be viable in practical applications, they must not only satisfy conventional mechanical property requirements but also exhibit a set of specific functional characteristics. Firstly, high ionic conductivity is essential to facilitate efficient ion transport, ensuring the stable and effective operation of ZIBs. In addition, hydrogel electrolytes must possess the capability to suppress the growth of zinc dendrites, which are a major cause of internal short circuits and associated safety hazards. By effectively inhibiting dendrite formation, hydrogel electrolytes can significantly improve both the safety and the operational lifespan of ZIBs.

#### Mechanical Property

The mechanical properties of hydrogel electrolytes are among their most distinguishing features compared to traditional aqueous electrolytes, conferring distinct advantages in a variety of applications. Specifically, hydrogel electrolytes exhibit high tensile strength, compressive strength, and flexibility, allowing them to endure external mechanical stresses without cracking or deformation. These mechanical characteristics enable hydrogel electrolytes to function reliably in complex operating environments, particularly in applications where flexibility and mechanical stability are essential, such as wearable devices, flexible electronics, and high-performance energy storage systems [34].

In terms of tensile strength—a key parameter indicating a material’s resistance to tensile failure, hydrogel electrolytes typically exhibit values ranging from 0.1 to 10 MPa. This strength is primarily attributed to chemical cross-linking (e.g., covalent bonds) and physical interactions (e.g., hydrogen bonding and ion coordination) between polymer chains. For instance, a boronic acid cross-linked polyvinyl alcohol/polyaniline (PVA/PANI) composite hydrogel demonstrates a tensile strength of up to 5.3 MPa, along with excellent ductility and fatigue resistance, maintaining structural integrity even after more than 1,000 bending cycles [35]. Such performance makes it well suited for the dynamic deformation requirements of flexible ZIBs.

Compressive strength is another critical parameter that reflects the hydrogel’s ability to withstand external pressure. In the context of ZIBs, compressive strength directly influences the structural stability and interfacial reliability of the electrolyte. Higher compressive strength helps preserve the dimensional stability of the hydrogel during repeated charge–discharge cycles, minimizing risks of interfacial delamination or cracking due to volume fluctuations, and thereby contributing to long-term cycling stability [36]. This property is closely related to the internal cross-linked network architecture of the hydrogel. Through optimization of the network design, both mechanical toughness and compressive resistance can be enhanced to better accommodate internal stress variations during battery operation.

Flexibility, typically characterized by elongation at break and elastic modulus, is another key indicator used to evaluate the deformation tolerance and adaptability of hydrogels. It is primarily governed by polymer chain entanglement and the dynamic breaking and reformation of reversible cross-links [37]. These dynamic bonding mechanisms allow hydrogels to dissipate mechanical energy efficiently, enabling them to accommodate complex deformations. In flexible energy storage devices, enhanced flexibility offers several advantages: It allows the hydrogel to conform intimately to the curved surfaces of electrodes, maintaining continuous ion transport pathways; moreover, high flexibility enables the hydrogel to withstand various mechanical deformations encountered during device assembly and operation, thereby ensuring stable electrochemical performance under diverse working conditions.

#### Ion Transporting Ability

The ion transport capability of hydrogel electrolytes is a critical parameter that significantly influences the electrochemical performance of ZIBs and has become a major focus of recent research. This capability is primarily governed by two key factors: ionic conductivity and the Zn^2+^ transference number (a parameter representing the fraction of ionic current carried specifically by Zn^2+^ ions in the electrolyte) [38, 39].

From the standpoint of ionic conductivity, this parameter serves as a direct measure of how efficiently ions migrate through the hydrogel electrolyte, thereby determining the overall rate of ion transport within the battery. Ionic conductivity is influenced by several interrelated factors, including ion concentration, polymer cross-linking structure, and water content [40].

Firstly, ion concentration dictates the number of mobile charge carriers per unit volume; higher concentrations increase the number of ions available for transport under an electric field, thus enhancing conductivity [25, 41]. Secondly, the cross-linking structure of the polymer network plays a crucial role. A moderate degree of cross-linking can maintain structural integrity while forming stable ionic pathways. However, excessive cross-linking may hinder the mobility of water molecules and ions, necessitating a balance between mechanical strength and ion mobility [42].

Among these parameters, water content is particularly influential. As the water content increases, the ionic conductivity of the hydrogel tends to improve significantly. This is because water molecules act as essential bridges within the polymer network, facilitating ion transport. An increase in water content expands ion-conductive channels, thereby enhancing the overall conductivity of the hydrogel.

The Zn^2+^ transference number, defined as the ratio of the migration rate of Zn^2+^ ions to the total migration rate of all ions in the electrolyte, is another important metric. In ZIBs, where Zn^2+^ serves as the primary charge carrier, a higher Zn^2+^ transference number indicates a greater contribution of Zn^2+^ to the ionic current during battery operation. This metric reflects the dominance of Zn^2+^ migration relative to other ionic species such as hydrated cations or free anions. A high Zn^2+^ transference number not only improves the utilization efficiency of active Zn^2+^ but also promotes a more uniform Zn^2+^ distribution on the anode surface, thereby reducing the risk of dendrite formation [43–45]. Moreover, enhanced Zn^2+^ transport efficiency shortens the ion diffusion path, enabling faster charge–discharge kinetics and improving rate capability. Additionally, a higher transference number minimizes the migration of free anions, helping to suppress undesirable side reactions such as corrosion and hydrogen evolution reactions (HER) [46].

In general, a Zn^2+^ transference number greater than approximately 0.5 indicates that most of the ionic current is carried by Zn^2+^ ions rather than by anions. This helps ensure uniform Zn deposition by minimizing ion concentration gradients near the anode surface, thereby suppressing dendrite formation. Moreover, tuning water content and cross-link density allows researchers to balance ionic conductivity and mechanical integrity: Increasing water enhances ion mobility but may weaken the network, whereas tighter cross-linking strengthens the gel yet restricts ion transport. Therefore, a rational compromise between these parameters is essential for achieving durable and efficient ZIBs.

#### Inhibit Dendrite Growth

The ability of hydrogel electrolytes to suppress zinc dendrite growth is critical for enhancing the cycling stability and safety of ZIBs [47]. Zinc dendrite formation is a major factor undermining the long-term reliability of ZIBs. It is typically initiated by uneven Zn^2+^ deposition on the anode surface, leading to local concentration gradients that promote dendrite nucleation and propagation [48, 49]. Hydrogel electrolytes can effectively mitigate dendrite formation through multiscale synergistic mechanisms, thereby significantly improving the overall electrochemical stability and operational safety of the batteries.

Firstly, the porous network structure and the presence of functional groups within hydrogel electrolytes help homogenize Zn^2+^ transport, thereby eliminating local concentration gradients and reducing the likelihood of non-uniform zinc deposition. For example, in the study by Li et al., Prussian blue analogues (PBs) were incorporated as functional fillers into the hydrogel electrolyte [50]. These PBs not only served as supplementary Zn^2+^ reservoirs but also constructed a 3D continuous ion diffusion network. The Lewis acidic metal centers in PBs promoted zinc salt dissociation, while the coordinated water molecules enhanced the Zn^2+^ transference number. These combined effects regulated Zn^2+^ migration pathways, alleviated local polarization, and facilitated uniform zinc deposition with effective dendrite suppression.

Secondly, functionalized hydrogels can further guide Zn^2+^ transport by introducing zinc-affinitive moieties, such as carboxyl, hydroxyl, amino, amide, and sulfonate groups, which promote uniform deposition even under high current densities [51]. For instance, Ma et al. developed a polyzwitterionic hydrogel composed of polyacrylamide (PAM), L-alanine (LA), and poly(2-(methacryloyloxy) ethyl) dimethyl-(3-sulfopropyl) ammonium hydroxide (PSBMA) [52]. This hydrogel incorporated –SO_3_^−^, –COO^−^, and C=O functional groups that not only formed strong coordination complexes with Zn^2+^ but also reconstructed its solvation shell, thereby significantly lowering the desolvation energy barrier. Theoretical calculations revealed a Zn^2+^-PSBMA coordination binding energy as high as 80.1 eV, effectively enabling directional Zn^2+^ deposition and highly uniform plating.

In addition to chemical regulation, the intrinsic physical properties of hydrogel electrolytes, such as high elasticity and self-healing capability, also contribute significantly to the dendrite suppression during charge–discharge cycling. For instance, Li et al. developed an ultrahigh-modulus hydrogel electrolyte composed of PVA, PAM, and polyacrylonitrile (PAN), achieving an exceptional elastic modulus of 198.5 MPa and toughness of 274.3 MJ m^−3^ [53]. This superior mechanical strength enabled the hydrogel to withstand localized stress from dendrite growth, thereby providing robust physical suppression. Notably, the hydrogel maintained approximately 70% water content and achieved a high ionic conductivity of 28.9 mS cm−1, ensuring both excellent ion transport and mechanical resilience.

Moreover, the self-healing capability of hydrogel electrolytes further mitigates dendrite formation. For example, Wang et al. designed a zinc alginate (ZA) hydrogel with dynamic and adaptive self-healing properties [54]. This hydrogel was capable of autonomously adjusting at the electrode–electrolyte interface to accommodate volumetric fluctuations and repair microcracks, thereby maintaining long-term structural and electrochemical stability throughout extended cycling.

In summary, the mechanical strength, ionic transport efficiency, and dendrite suppression ability of hydrogel electrolytes are strongly influenced by the cross-linking strategy of the polymer network. Different cross-linking mechanisms exhibit significant differences and trade-offs in performance. For instance, chemical cross-linking based on covalent bonds can form a robust three-dimensional network, providing excellent mechanical modulus and long-term stability, which is crucial for physically blocking dendrites. However, its rigid network may restrict the movement of chain segments, thereby sacrificing some ionic conductivity and self-healing ability. On the contrary, physical cross-linking that relies on hydrogen bonds, ion coordination, and other interactions has dynamic reversible characteristics. It not only endows materials with self-healing properties and environmental responsiveness, but also its looser network structure is more conducive to ion migration. However, its mechanical strength and stability are usually poor, and it is prone to network disintegration under extreme conditions (such as high temperature and dehydration). Therefore, hybrid systems that combine these two methods are increasingly being used to balance tensile strength, compressive resistance, and flexibility, enabling hydrogel electrolytes to meet the demanding requirements of ZIBs.

### Design Requirements

Building upon the fundamental characteristics outlined above, this section establishes how these intrinsic material properties can be translated into explicit design criteria for practical zinc-ion battery applications. In this context, the design requirements are not merely descriptions of material attributes, but rather performance-oriented benchmarks that guide the optimization of hydrogel electrolytes toward reliable, safe, and scalable operation.

**Fig. 2:**
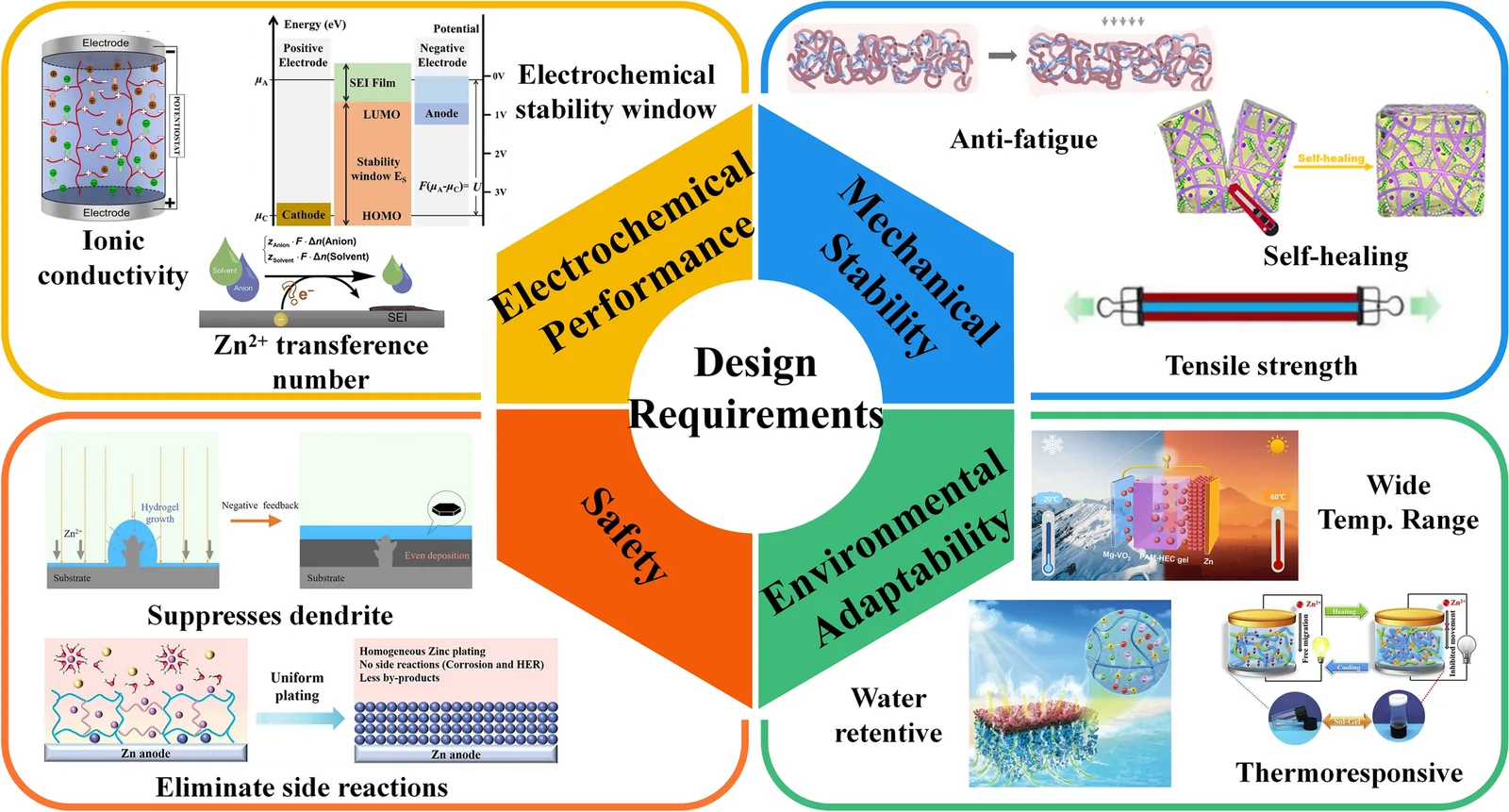
Schematic illustration of the essential design requirements for ZIBs. The images on electrochemical performance of ZIBs are reproduced with permission [56–58]. Copyright 2018, American Chemical Society, Copyright 2022, Springer Nature, Copyright 2022, Elsevier. The images on mechanical stability of ZIBs are reproduced with permission [59–61]. Copyright 2025, Wiley, Copyright 2023, Wiley, Copyright 2021, Elsevier. The images on safety of ZIBs are reproduced with permission [62, 62]. Copyright 2024, Wiley, Copyright 2024, Elsevier. The images on environmental adaptability of ZIBs are reproduced with permission [64–66]. Copyright 2024, American Chemical Society, Copyright 2023, Wiley, Copyright 2018, Elsevier

After clarifying the core functional requirements of hydrogel electrolytes, their practical application performance must be systematically evaluated using multiple key performance indicators (Fig. 2). These indicators encompass not only electrochemical parameters, such as ionic conductivity and Zn^2+^ transference number, but also mechanical attributes, including tensile strength, flexibility, and interfacial compatibility with electrodes [55].

Furthermore, as ZIBs evolve toward higher safety standards, longer cycle life, and lower manufacturing costs, the design of hydrogel electrolytes must also incorporate considerations of safety and environmental adaptability, particularly tolerance to variations in temperature and humidity.

To meet these comprehensive performance requirements, the development of hydrogel electrolytes should adopt a multidimensional design strategy. This includes the optimization of electrochemical properties, enhancement of mechanical robustness, assurance of operational safety, and improvement of environmental resilience, each of which is essential for ensuring the reliable and scalable application of hydrogel-based electrolytes in next-generation ZIBs.

#### Electrochemical Performance Requirements

The electrochemical performance of hydrogel electrolytes is a critical determinant of their practical applicability and efficiency in ZIBs and is primarily evaluated through parameters such as ionic conductivity, Zn^2+^ transference number, and electrochemical stability window (ESW, the voltage range within which the electrolyte is neither oxidized nor reduced). An ideal hydrogel electrolyte should enable continuous and uniform ion transport while maintaining long-term stability within the electrochemical window, thereby minimizing the occurrence of side reactions such as hydrogen evolution. Together, these parameters determine how efficiently and safely charge is transferred within the cell, providing a quantitative foundation for evaluating electrolyte performance in practical ZIB systems.

First, high ionic conductivity is essential for facilitating efficient Zn^2+^ migration within the electrolyte, reducing internal resistance, and ultimately enhancing both the rate capability and the overall energy output of the battery [45]. Achieving this requires the construction of interconnected ion-conductive pathways and the optimization of polymer network architecture and water content to promote rapid ion transport.

Second, a reasonably high Zn^2+^ transference number is equally important, as it improves charge transport efficiency, alleviates concentration polarization, and effectively suppresses zinc dendrite formation [44]. This can be accomplished by incorporating functional groups with selective coordination affinity for Zn^2+^ or by engineering directional ionic transport channels within the hydrogel matrix.

In addition, the hydrogel electrolyte must possess a wide ESW to prevent electrolyte decomposition within the operational voltage range, particularly to suppress hydrogen evolution [67]. Enhancing this stability typically involves strategies such as reducing the free water content, tuning the pH of the electrolyte, and incorporating electrochemical stabilizers to improve the system’s resistance to degradation under applied electric fields.

It is worth noting that trade-offs often exist in hydrogel electrolytes. On the one hand, enhancing ionic conductivity, for example, by raising water content or lowering cross-linking density, can compromise mechanical robustness and structural stability. On the other hand, water content also governs the balance between overall conductivity and Zn^2+^ transference number: Excess free water improves ion mobility but reduces the relative contribution of Zn^2+^, while limiting free water increases selective Zn^2+^ transport yet may hinder ion conduction. Therefore, balancing ionic transport with mechanical integrity has become a key design principle. This balance can be achieved through representative strategies, including dual-network architectures, nanostructured fillers, and dynamic cross-linking chemistries, which together enable both efficient ion transport and robust mechanical support.

#### Mechanical Stability Requirements

During long-term operation of ZIBs, repeated charge–discharge cycles induce continuous volumetric expansion and contraction of the electrodes [68]. This dynamic behavior imposes stringent requirements on the structural stability of the electrolyte material. As the critical interfacial medium between the electrodes and the electrolyte, hydrogel electrolytes must possess robust mechanical properties to maintain interfacial integrity and extend battery cycle life [69].

An ideal hydrogel electrolyte should exhibit excellent structural resilience and shape-recovery capability, enabling it to rapidly restore its original form after deformation or external mechanical stress. This prevents issues such as cracking, fracture, or delamination from the electrode surface, which could otherwise interrupt ionic transport pathways or compromise interfacial contact, ultimately leading to capacity fading or device failure [68]. To address these challenges, material design must consider key mechanical parameters such as tensile strength, elongation at break, and fatigue resistance [70]. These properties can be effectively enhanced by optimizing the polymer network architecture, tuning the cross-linking density, or incorporating flexible reinforcing components.

Moreover, adequate flexibility and interfacial adhesion are essential to ensure mechanical compatibility with a variety of device configurations, particularly in flexible and wearable batteries. Such mechanical robustness is fundamental for sustaining long-term electrochemical performance under complex and dynamically changing operating conditions.

#### Safety Requirements

In the practical application of ZIBs, safety remains a paramount concern, with the electrolyte playing a central role in defining the system’s safety boundaries. As the component in direct contact with both electrodes, the hydrogel electrolyte critically influences thermal and electrochemical stability, as well as the suppression of undesirable side reactions, all of which are essential for ensuring the safe operation of ZIBs.

On the one hand, the hydrogel electrolyte must exhibit excellent thermal stability, maintaining its structural integrity and functional performance across a broad temperature range. This prevents issues such as melting, decomposition, or gas evolution under elevated temperatures. More importantly, the electrolyte should effectively suppress the nucleation and growth of zinc dendrites on the anode surface [53, 71]. If left uncontrolled, dendrites can penetrate the separator and cause internal short circuits, potentially leading to thermal runaway and catastrophic failure. To mitigate this, hydrogel electrolytes must be engineered to regulate ion flux distribution and promote uniform Zn^2+^ deposition, thereby offering both physical barriers and chemical inhibition mechanisms to impede dendrite propagation [43].

In addition, careful control of the free water content within the hydrogel is essential to minimize HER. Excessive gas generation not only increases internal pressure but also poses a serious safety risk due to the potential for cell rupture or explosion [72]. Therefore, the design of safe hydrogel electrolytes requires comprehensive optimization strategies encompassing material thermal stability, interfacial ion regulation, and suppression of parasitic reactions, ultimately ensuring the safety and reliability of ZIB systems during long-term operation.

#### Environmental Adaptability

ZIBs hold significant promise for future energy storage applications, particularly in areas such as flexible electronics, portable devices, and large-scale energy storage systems, where stable operation under diverse and often extreme environmental conditions is essential. Consequently, the environmental adaptability of hydrogel electrolytes has become a critical factor in assessing their practical viability.

To function effectively, hydrogel electrolytes must retain their structural integrity and electrochemical stability under challenging conditions, including extreme temperatures, high humidity, and dry environments [73, 74]. At low temperatures, water within the hydrogel matrix may freeze, leading to a dramatic reduction in ionic conductivity. To address this issue, strategies such as tuning the polymer cross-linking density and incorporating antifreeze agents have been employed to improve frost resistance. Conversely, under high-temperature or arid conditions, hydrogels are prone to water evaporation, volume shrinkage, and potential structural collapse, all of which can compromise the interfacial stability with the electrodes.

To mitigate these thermal and dehydration-related challenges, several approaches have been proposed, including the use of water-retentive polymer matrices, the incorporation of hydrophilic fillers, and the construction of polymer networks stabilized by multiple hydrogen bonding interactions [75]. These strategies effectively enhance both water retention and thermal resilience.

Furthermore, hydrogel electrolytes must also exhibit sufficient environmental tolerance and processing compatibility during practical manufacturing and device integration. Their ability to withstand environmental stress while accommodating various fabrication techniques and device architectures is essential for enabling scalable production and deployment.

Furthermore, hydrogel electrolytes must also exhibit sufficient environmental tolerance and processing compatibility during practical manufacturing and device integration. Their ability to withstand environmental stress while accommodating various fabrication techniques is essential for enabling scalable production and deployment. Beyond operational conditions, environmental adaptability should also be considered from a sustainability perspective. Natural polymer-based hydrogels not only provide favorable ion transport and interfacial properties, but also offer biodegradability and renewability, thereby reducing ecological burden and enhancing safety in wearable or implantable applications. Nevertheless, careful design is required to balance biodegradability with long-term electrochemical stability, ensuring that eco-friendly materials can meet the durability demands of practical ZIB systems.

## Composition and Design Strategy of Hydrogel Electrolyte

Having clarified the performance requirements, the next step is to understand how material composition and structural design translate these criteria into real functional systems. In the preceding section, the key performance requirements of hydrogel electrolytes for ZIBs, including electrochemical performance, mechanical stability, safety, and environmental adaptability, were systematically reviewed. To fulfill these demanding criteria, extensive research efforts have been dedicated to material selection, component regulation, and fabrication strategies [76]. It is important to note that the performance of hydrogel electrolytes is not an intrinsic material property, but rather the result of synergistic control over their chemical composition, structural design, and processing methods [77, 78].

In recent years, considerable progress has been achieved in optimizing various facets of hydrogel electrolytes. These include the selection and functional modification of polymer matrices, incorporation of performance-enhancing additives, construction of efficient ion transport channels, refinement of cross-linking strategies, and interface engineering between the electrolyte and electrodes. Each of these factors, whether related to the type of polymer backbone, the nature of functional fillers, the cross-linking mechanism, or the tuning of electrolyte composition, plays a pivotal role in achieving targeted enhancements in one or more critical performance metrics.

### Selection of Substrate Materials

The fundamental framework of hydrogel electrolytes is predominantly composed of a polymeric network, and thus, the choice of substrate material plays a decisive role in determining critical performance attributes, including mechanical strength, water retention capacity, ionic transport efficiency, and interfacial stability with electrodes [79]. An ideal polymer matrix should exhibit not only high hydrophilicity and ionic conductivity but also a well-balanced combination of flexibility, mechanical robustness, and chemical stability across the operational conditions of ZIBs.

Currently, hydrogel matrix materials are generally classified into three categories: natural polymers, synthetic polymers, and polymer composites [80]. Each class offers distinct advantages and limitations. Natural polymers often feature excellent biocompatibility and environmental friendliness, whereas synthetic polymers allow for tunable structures and tailored properties. Composite systems aim to combine the merits of both, providing enhanced performance through synergistic effects. Researchers typically select or engineer these materials in a targeted manner, based on specific performance requirements and application scenarios in ZIBs.

#### Natural Polymer

Natural polymer materials, such as gelatin, sodium alginate (SA), carboxymethyl cellulose (CMC), and chitosan, have attracted significant attention due to their inherent biodegradability, excellent hydrophilicity, and low cost [81]. These polymers are rich in functional groups, including –OH, –COOH, and C=O moieties, which play a critical role in suppressing side reactions (e.g., HER) and mitigating corrosion of the Zn anode in ZIBs [82].

**Fig. 3:**
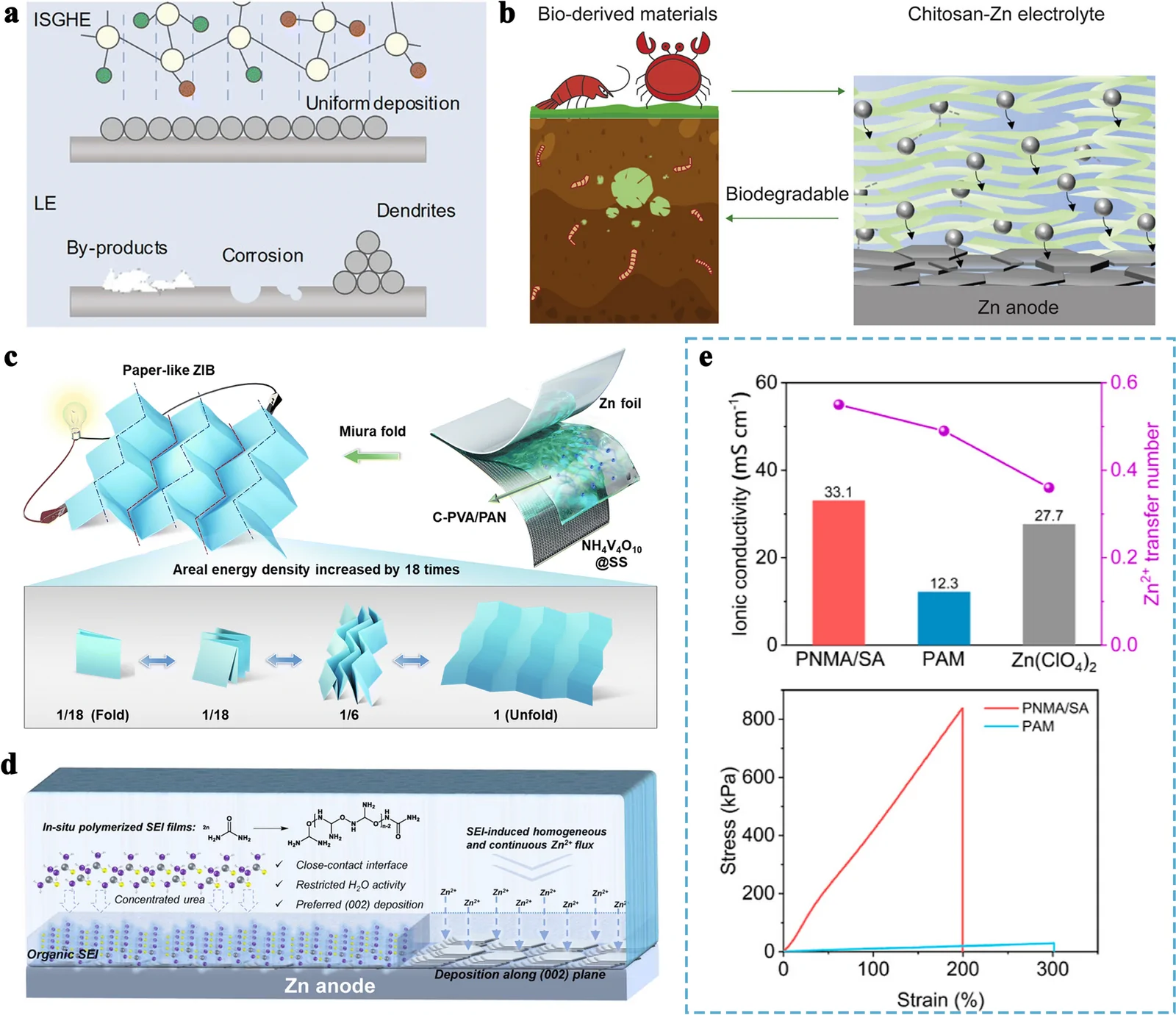
**a** Schematic illustration of Zn anodes cycled in the hydrogel electrolyte (ISGHE) and liquid electrolyte (LE). Reproduced with permission [83]. Copyright 2021, The Royal Society of Chemistry. **b** Illustration highlighting the role of chitosan biomaterials in constructing eco-friendly ZIBs. Reproduced with permission [84]. Copyright 2022, Elsevier. **c** Schematic illustration of the preparation for hydrogel and paper-like ZIBs. Reproduced with permission [85]. Copyright 2024, The Royal Society of Chemistry. **d** Schematic illustration of the underlying mechanism responsible for the improved electrochemical performance achieved with gel. Reproduced with permission [86]. Copyright 2024, Wiley. **e** Ionic conductivity of PNMA/SA, PAM, and Zn(ClO_4_)_2_ electrolytes, along with the corresponding stress–strain curves of PNMA/SA and PAM gels. Reproduced with permission [87]. Copyright 2024, American Chemical Society

For example, Zhou et al. developed a composite hydrogel electrolyte based on natural biomacromolecules, iota-carrageenan (IC), and SA, for high-performance ZIBs [83]. Their study demonstrated that the abundant –COO^−^ and –SO_3_^−^ groups in these natural polymers not only formed stable coordination complexes with Zn^2+^ ions but also established dense hydrogen bonding networks that effectively immobilized free water molecules. This dual mechanism significantly suppressed hydrogen evolution and by-product formation, thereby improving both the interfacial stability and reversibility of the Zn anode (Fig. 3a).

In another study, Hu et al. designed a sustainable chitosan-Zn composite hydrogel electrolyte for use in high-rate ZIBs [84]. By leveraging the coordination interactions between Zn^2+^ and the –NH_2_ and –OH groups in chitosan, and fine-tuning the water content via mechanical compression, a compact and highly conductive ion transport network was constructed. The resulting hydrogel exhibited a remarkable ionic conductivity of 71.8 mS cm^−1^. Moreover, its abundant functional groups directed zinc deposition into parallel hexagonal nanosheet structures, thereby reducing the active interfacial area between the electrolyte and the Zn anode and suppressing interfacial side reactions (Fig. 3b).

#### Synthetic Polymer

Common synthetic polymers, such as PVA, PAN, PAM, and polyacrylic acid (PAA), offer distinct advantages in terms of structural tunability, batch-to-batch reproducibility, and superior mechanical performance, making them ideal candidates for 3D elastic and ductile hydrogel networks [88, 89]. Moreover, the hydrophilicity, adhesion, and electrochemical stability of these materials can be precisely regulated through molecular design and functional modification, which has become a major focus in recent hydrogel electrolyte research.

For instance, Sun et al. employed a hydrophobic and rigid PAN network to constrain the swelling behavior of a hydrophilic PVA network, thereby forming a mechanically stable double-network hydrogel electrolyte [85]. This design not only improved interfacial adhesion and the uniformity of Zn^2+^ deposition through regulation of cross-link density and functional group distribution, but also imparted the electrolyte with excellent mechanical strength and flexibility. As a result, the hydrogel was well suited for paper-like ZIBs operating under complex mechanical deformation scenarios, such as folding and compression (Fig. 3c).

In another study, Li et al. introduced urea molecules featuring both hydrogen bond donor and acceptor functionalities into a PVA matrix to effectively mitigate the “salting-out effect” of ZnSO_4_ in hydrogel systems [86]. This molecular engineering strategy reconstructed the hydrogen bonding microenvironment between PVA and water, significantly enhancing the zinc salt retention capacity. Through a molecular bridging effect, the urea molecules disentangled aggregated PVA chains, thereby forming continuous and smooth Zn^2+^ migration pathways. Simultaneously, urea molecules migrated toward and adsorbed onto the zinc surface, facilitating the formation of a stable transference number layer during electrochemical cycling. This contributed to uniform Zn deposition and stabilized the electrode–electrolyte interface, ultimately improving both the electrochemical performance and long-term durability of the battery (Fig. 3d).

#### Composite Polymer

To integrate the respective advantages of natural and synthetic polymers, composite polymer matrix systems have emerged as a major focus in the design of hydrogel electrolytes. The synergistic combination of natural and synthetic components effectively addresses the inherent performance limitations of single-component systems. Natural polymers, owing to their abundant functional groups and excellent biocompatibility, provide efficient ion transport sites, suppress undesirable side reactions, and facilitate uniform Zn^2+^ deposition. In contrast, synthetic polymers contribute superior mechanical strength, structural tunability, and enhanced processing stability, thereby improving the overall mechanical robustness and environmental adaptability of the hydrogel.

This composite strategy not only optimizes ion transport pathways and enhances ionic conductivity but also effectively mitigates zinc dendrite formation and extends battery cycling life. For example, Du et al. developed a novel dual-network hydrogel electrolyte (PPZ) by combining the natural polymer pullulan (PuL) with PVA [90]. The incorporation of PuL significantly improved the tensile strength and adhesive properties of the hydrogel, while its abundant –OH groups served as binding sites for H_2_O molecules, enhancing water retention. Simultaneously, PVA contributed to the mechanical stability and structural integrity of the network. The resulting PPZ hydrogel electrolyte demonstrated excellent cycling stability and electrochemical performance in flexible ZIBs.

In another study, Lv et al. designed a gel electrolyte based on a dual-network structure composed of poly(N-methylolacrylamide) (PNMA) and sodium alginate (SA), further highlighting the advantages of hybrid polymers [87]. As a natural polymer, SA is rich in –COO^−^ and –OH functional groups, which offer numerous coordination sites for Zn^2+^ and facilitate fast ion migration by modulating the Zn^2+^ solvation structure. Meanwhile, PNMA undergoes self-cross-linking to form a dense polymer network, imparting excellent mechanical strength and water retention capability. The resulting gel electrolyte achieved an impressive ionic conductivity of 33.1 mS cm^−1^ and a tensile strength of 838 kPa, significantly outperforming conventional single-network hydrogels (Fig. 3e). Moreover, it maintained electrochemical stability over 2,000 h of cycling in symmetric cells and effectively suppressed dendrite growth and parasitic reactions, demonstrating great potential for application in high-performance flexible ZIBs.

Beyond these examples, very recent studies have further highlighted the potential of double-network composite hydrogels. For instance, Wang et al. constructed an agarose–polyacrylamide (AG-PAM) double-network hydrogel as a protective interface for Zn anodes, which not only facilitated Zn^2+^ desolvation and homogenized ion flux but also enabled dendrite-free deposition, achieving ultralong cycling stability of over 3,500 h in symmetric cells [91]. Similarly, Unruangsri et al. developed a CO_2_-derived polycarbonate/PAM double-network hydrogel, where sulfonate-functionalized polycarbonate introduced additional Zn^2+^-coordination channels and mechanical reinforcement [92]. This system delivered a Zn^2+^ transference number of 0.75 and demonstrated superior cycling stability with 66.7% capacity retention after 1,200 cycles. These results confirm that composite double-network hydrogels can simultaneously enhance mechanical robustness, ion transport, and interfacial stability, outperforming conventional single-network systems. To further clarify the similarities and differences among these three categories, their representative features are summarized in Table 1 in terms of advantages, limitations, mechanical properties, and processability. Furthermore, representative electrochemical and physical parameters of typical hydrogel electrolytes are compared in Table 2 to provide a quantitative insight into the performance differences among natural, synthetic, and composite polymer matrices.

**Table 1:** Comparison of natural, synthetic, and composite polymer matrices for hydrogel electrolytes

Category	Representative materials	Mechanical properties	Processability	Processability/cost	Advantages	Limitations
Natural polymers	Cellulose, gelatin, chitosan, sodium alginate, guar gum, etc	Generally limited mechanical robustness	Renewable and biodegradable	Abundant, renewable, low cost	Sustainable origin, intrinsic biocompatibility, abundant functional groups for Zn^2+^ coordination	Poor stretchability, limited long-term stability
Synthetic polymers	PAM, PAA, PANa, PVA, PVDF, etc	Superior mechanical strength and flexibility	Amenable to large-scale fabrication	High designability, large-scale production, cost varies	Structural tunability, reproducibility, adjustable hydrophilicity/adhesion	Certain monomers suffer from high cost and low ionic conductivity
Composite systems	Pullulan–PVA, PNMA–SA, etc	Strong mechanical strength	Requiring multi-component blending with stringent ratio control	Processability depends on filler; sometimes complex preparation	Synergistically integrates merits of natural and synthetic matrices	Fabrication complexity; reproducibility challenges

**Table 2:** Comparative summary of hydrogel electrolytes based on natural, synthetic, and composite polymer matrices

Category	Representative System	Ionic conductivity (mS cm^−1^)	Zn^2+^ transference number	Mechanical properties	Water retention (25 °C, 50% RH, 24 h)	Operating temperature (°C)	Refs
Natural polymers	Iota-carrageenan/sodium alginate	58.9	0.58	–	–	RT	[83]
	Chitosan	72	–	Tensile strength of 7.4 MPa	–	RT	[84]
	Hyaluronic acid	47.7	0.73	Compressive strength of 0.18 MPa	–	0 ~ 40	[93]
	Gelatin/*β*-cyclodextrin	24.89	0.49	Tensile strength of 0.52 MPa	–	0 ~ 37	[94]
Synthetic polymers	PAN/PVA	17.4	0.64	Tensile strength of 24.7 MPa	25% retention (120 h, 25 °C)	25 ~ 60	[85]
	PVA/PAM/PAN	28.9	0.52	Fracture strength of 24.8 MPa	–	RT	[53]
	P(AM-co-AA)	60.6	0.88	Compressive strength of 11.3 MPa	–	RT	[95]
Composite systems	PAM/*β*-cyclodextrin	27.8	0.64	Elongation of 707%	83.5% retention (48 h, 25 °C)	–40 ~ 25	[38]
	PAM/lignin-containing cellulose nanofibers	21.57	0.79	Tensile strength of 0.35 MPa	76% retention (144 h, 25 °C)	RT	[96]

Room temperature (RT)

### Electrolyte Component Optimization

Beyond the selection of polymer matrix materials, the regulation of functional components within the hydrogel electrolyte is equally critical to achieving optimal performance. A typical hydrogel electrolyte consists of a polymer network, water or solvent systems, and a variety of auxiliary additives [97]. Through the precise tuning of these components, key properties such as ionic conductivity, electrochemical stability, interfacial compatibility, and overall safety can be significantly enhanced.

Each constituent in the hydrogel system fulfills a specific functional role, ranging from facilitating efficient ion transport, suppressing parasitic side reactions, modulating interfacial behavior, to improving environmental adaptability. The coordinated optimization of these components enables the development of high-performance electrolytes tailored to diverse application scenarios.

The following section provides a comprehensive overview of representative strategies for component regulation, summarizing recent advances and practical approaches employed to improve the overall functionality of hydrogel electrolytes.

#### Regulation of Zinc Salt Type and Concentration

Zinc salts, as the primary source of Zn^2+^ ions, are fundamental ionic components in hydrogel electrolytes. Commonly utilized zinc salts include ZnSO_4_, ZnCl_2_, Zn(ClO_4_)_2_, Zn(TFSI)_2_, Zn(CF_3_SO_3_)_2_, and Zn(CH_3_COO)_2_ [98]. The type of zinc salt critically influences the electrolyte’s ionic conductivity, ESW, and interfacial behavior with electrodes. In addition, zinc salt concentration modulates the hydration structure of Zn^2+^ and its transference number, thereby offering an effective pathway for optimizing ion transport mechanism and electrolyte stability.

From a practical standpoint, selecting the appropriate zinc salt is essential for enhancing the overall performance of ZIBs. For instance, ZnSO_4_ is widely used due to its low cost, high electrochemical stability, and good compatibility with zinc metal [99]. However, for applications requiring higher ionic conductivity, salts with large, weakly coordinating anions, such as Zn(CF_3_SO_3_)_2_ and Zn(TFSI)_2_, are more favorable [100]. These bulky anions exhibit weaker electrostatic interactions with Zn^2+^, resulting in a higher concentration of free Zn^2+^ ions, increased Zn^2+^ transference number, and improved overall ionic conductivity. Although ZnCl_2_ offers high conductivity, its narrow ESW limits its practical utility [101].

The ESW is a key determinant of battery safety and energy density. According to frontier orbital theory, the ESW is governed by the relative energy levels of the anode (*μ*_A_), cathode (*μ*_C_), and the electrolyte’s lowest unoccupied molecular orbital (LUMO) and highest occupied molecular orbital (HOMO) [102]. Strategies to widen this window include elevating the LUMO level to suppress HER or lowering the HOMO level to delay oxygen evolution reactions (OER) [56]. For example, the CF_3_SO_3_^−^ anion, due to its large ionic radius and low charge density, reduces the activity of water molecules coordinated with Zn^2+^, thereby effectively broadening the ESW.

Zinc salts also play a pivotal role in regulating interfacial behavior during battery operation. At the electrolyte/Zn interface, different salts induce the formation of distinct SEI layers, which affect electrode reaction kinetics and long-term cycling stability. For example, ZnSO_4_ can facilitate the reversible formation and dissolution of Zn_4_(OH)_6_SO_4_·5H_2_O on the zinc anode surface, promoting rapid Zn^2+^ plating/stripping kinetics while minimizing corrosion and dendrite growth [103]. The addition of specific additives further assists in modulating SEI composition, thereby enhancing interfacial stability and extending cycle life [104].

Moreover, zinc salt concentration significantly impacts the hydration structure of Zn^2+^ ions and their transference number [105]. At low concentrations, Zn^2+^ tends to form highly hydrated complexes with large ionic radii, resulting in high migration resistance and a low transference number. Increasing the salt concentration reduces the number of coordinated water molecules, thus decreasing the effective ionic radius and enhancing the transference number. However, excessive salt concentrations may lead to the formation of ionic aggregates or clusters, which hinder ion mobility, lower ionic conductivity, and reduce diffusion coefficients [106]. Therefore, precise control over zinc salt concentration is crucial to optimizing the ion transport environment, improving conductivity and electrolyte stability, and minimizing degradation during cycling.

However, salt concentration inherently involves multiple trade-offs. On the one hand, increasing concentration reduces free water activity and suppresses side reactions, thereby broadening the ESW and improving interfacial compatibility. On the other hand, high-concentration systems usually suffer from increased viscosity, and reduced ion mobility, which may compromise rate capability and long-term cycling performance.

Conversely, dilute electrolytes provide lower viscosity and facile ion transport, but their excessive water content tends to narrow ESW and accelerate parasitic reactions. An appropriately balanced concentration also reduces water evaporation and electrolyte decomposition, thereby enhancing the overall safety and reliability of ZIBs.

#### Additives

In ZIBs, the overall performance of hydrogel electrolytes plays a decisive role in determining both cycle life and operational safety. The incorporation of functional additives enables precise regulation of the hydrogel’s microstructure, solvation environment, and interfacial behavior, thereby facilitating multidimensional performance enhancement.

**Fig. 4:**
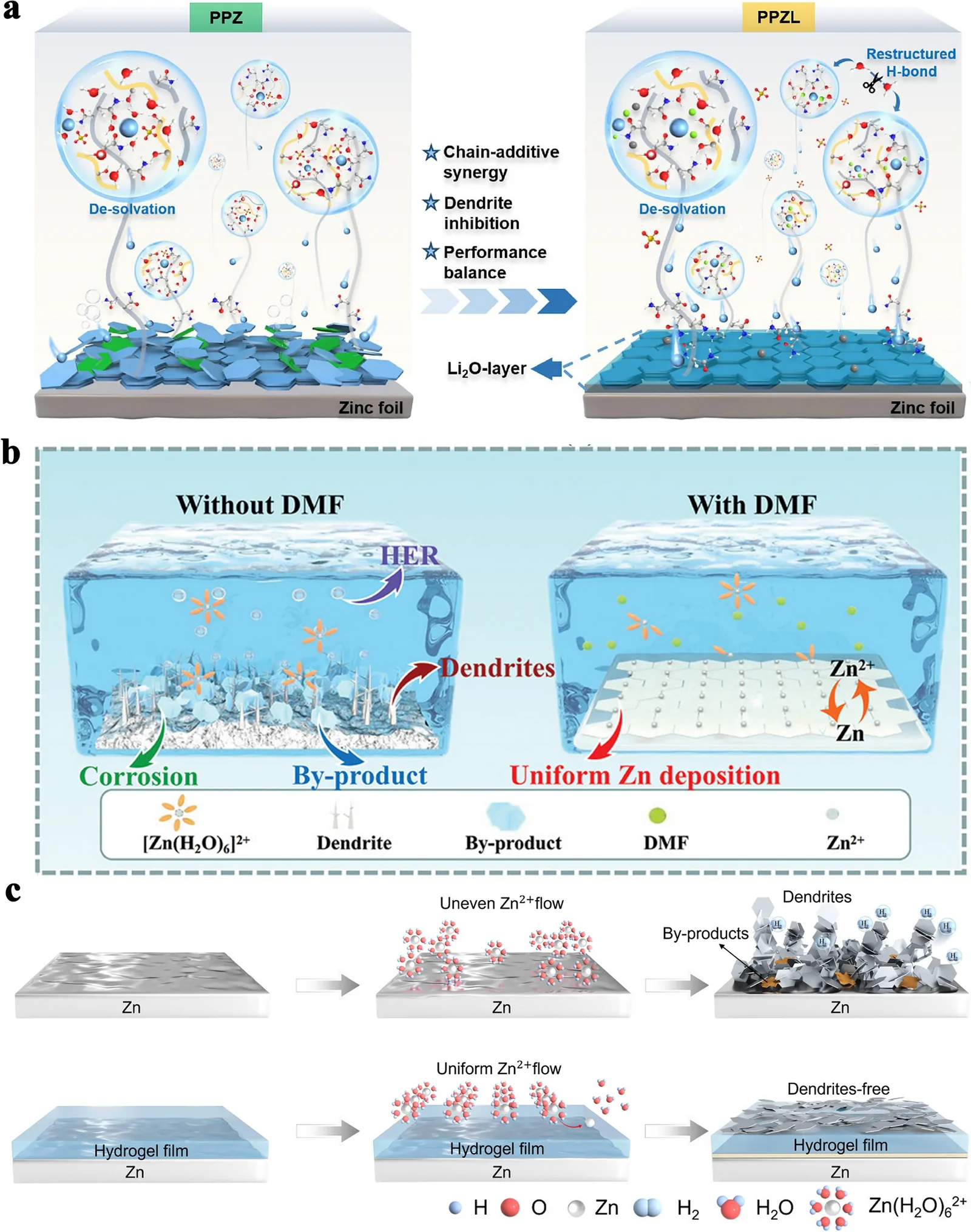
**a** Schematic illustration of the design concept and optimization strategy for PPZL hydrogel electrolytes [108]. Copyright 2025, Elsevier. **b** Comparative illustration showing the evolution of Zn anodes in hydrogel electrolytes in the presence and absence of DMF. Reproduced with permission [69]. Copyright 2024, Wiley. **c** Schematic illustration comparing Zn^2+^ deposition on bare Zn and MXene-CNF|Zn composite anodes. Reproduced with permission [109]. Copyright 2025, American Chemical Society

For instance, metal cation additives (e.g., Li^+^, Na^+^, Mg^2+^) can significantly suppress parasitic reactions through mechanisms such as electrostatic shielding and solvation structure modulation [19, 107]. Zhang et al. introduced 2 M LiCl into a dual-network PAM/PVA/ZnSO_4_ (PPZ) hydrogel to form a modified electrolyte (PPZL) [108]. This chain-additive synergistic design allowed for comprehensive interfacial optimization: functional groups on the PAM chains interacted with Cl^−^, Zn^2+^, and H_2_O to reconstruct the Zn^2+^ solvation environment, while Li^+^ contributed to the in-situ formation of a Li_2_O-based SEI layer at the Zn anode interface. This dual mechanism effectively suppressed parasitic side reactions (Fig. 4a), reduced the polarization voltage of Zn||Zn symmetric cells from 272 mV (with PPZ) to 98 mV at 5 mA cm^−2^, and enabled an ultralong cycling life of 8,000 h at 1 mA cm^−2^. Additionally, a zinc-ion hybrid capacitor assembled with this system achieved 98.5% capacity retention after 100,000 cycles at a high current density of 20 A g^−1^, demonstrating the synergistic benefits of dual-network hydrogels and functional additive engineering.

To further regulate Zn^2+^ transport behavior, chelating agents such as N,N-dimethylformamide (DMF), citric acid (CA), ethylenediaminetetraacetic acid (EDTA), and amino acids have been introduced to form reversible coordination interactions with Zn^2+^. These agents reduce free water activity, construct ordered ion migration pathways, and guide uniform Zn deposition through crystal facet-selective adsorption [110]. For instance, Qian et al. incorporated the polar aprotic solvent DMF into a PAM-based hydrogel electrolyte [69]. The strong coordination between the carbonyl group of DMF and Zn^2+^ facilitated the formation of directional ion transport channels (Fig. 4b). Density functional theory (DFT) calculations showed that DMF exhibited stronger adsorption energy on the Zn (002) crystal plane compared to water molecules, thereby promoting parallel Zn^2+^ stacking and dense, dendrite-free metal deposition. Furthermore, synergistic interaction between DMF and the polymer matrix disrupted the hydrogen bonding network among water molecules, improving the electrolyte’s temperature adaptability and structural stability.

In addition to molecular additives, two-dimensional materials (e.g., MXene, graphene oxide) are increasingly employed to enhance the structural integrity of the hydrogel network and improve the orderliness of ion transport pathways [111]. Liu et al. developed a dual-network hydrogel interfacial layer comprising 2D MXene and 1D cellulose nanofibers (CNFs), which markedly improved zinc anode cycling stability [109]. The MXene-CNF hydrogel exhibited excellent mechanical properties along with dual conductivity, featuring an electronic conductivity of 1.53 S cm^−1^ and an ionic conductivity of 0.52 mS cm^−1^. This architecture provided an ideal microenvironment for uniform Zn^2+^ migration. Notably, zincophilic functional groups on MXene surfaces, combined with the mechanically supportive CNF cross-linked network, enabled the formation of an interfacial layer with coupled multifield effects. This included stress confinement and homogeneous ion/electron field distribution across the Zn surface, effectively suppressing dendrite formation and parasitic reactions (Fig. 4c).

These findings highlight the importance of nano-engineering strategies. In particular, nanostructural modifications involving carbon nanotubes, graphene oxide, MXenes, and inorganic nanoparticles have shown great potential to further tailor the mechanical stability, ionic conductivity, and thermal stability of hydrogel electrolytes. Their high surface area and abundant functional groups enable strong interfacial interactions with polymer chains, thereby reinforcing the network and enhancing mechanical stability. Conductive nanostructures further establish continuous ion/electron transport channels, which improve ionic conductivity and Zn^2+^ deposition uniformity. Meanwhile, inorganic nanoparticles act as physical fillers that stabilize the hydrogel matrix and mitigate water evaporation, contributing to enhanced thermal stability. Overall, nano-engineering is not only a means of reinforcing polymer networks but also a powerful strategy to couple multiple functionalities within hydrogel electrolytes.

## Functional Optimization Strategy of Hydrogel Electrolyte

With the continuous advancement of hydrogel electrolyte fabrication technologies, traditional approaches focused solely on physical structure optimization are no longer sufficient to meet the increasingly multifaceted performance demands of high-performance ZIBs, including energy density, cycle life, and operational stability. As such, beyond material composition and network design, expanding the functional capabilities of hydrogel electrolytes has become a critical direction for achieving next-generation battery performance.

Functional optimization strategies involve the deliberate integration of adaptive or responsive features into the electrolyte system, going beyond baseline electrochemical performance to ensure stability and resilience under extreme or dynamic operating conditions. These advanced functionalities include wide-temperature-range adaptability, self-healing ability, anti-freezing and high-temperature resistance, anti-drying performance, flame retardancy, and environmental responsiveness. The incorporation of such features not only improves the operational reliability and longevity of battery systems but also enables their application in demanding scenarios such as flexible and wearable electronics, low-temperature environments, high-humidity conditions, and safety–critical settings [112, 113].

Therefore, this section focuses on several representative functional optimization strategies for hydrogel electrolytes. The discussion will highlight key advances in anti-freezing performance, high-temperature stability, thermoresponsive behavior, self-healing properties, and intrinsic safety enhancements, each of which contributes to the development of robust, multifunctional electrolyte systems for next-generation ZIBs.

### Anti-Freezing Performance

Water molecules in hydrogels electrolytes can be broadly classified into free water, weakly bound water, and unfrozen water, depending on their interaction strength with the polymer network. Under harsh cold conditions, the freezing of free water can lead to a significant reduction in mechanical integrity, hindered ion transport, and diminished ionic conductivity, potentially resulting in complete device failure [114]. This limitation severely restricts the practical applicability of hydrogel-based ZIBs in outdoor or extreme climatic environments. To address this challenge, various anti-freezing strategies have been developed, primarily focusing on the incorporation of organic solvents and high-concentration salt solutions to suppress ice formation while preserving the flexibility and conductivity of the hydrogel at subzero temperatures.

One effective approach involves the introduction of organic solvents, which disrupt the hydrogen bonding network of water molecules. These solvents form strong hydrogen bonds with water, thereby lowering the freezing point and preventing crystallization. Additionally, many organic solvents can coordinate with Zn^2+^ ions, modulating their solvation structure to reduce side reactions and inhibit dendrite growth [115]. Commonly used anti-freezing agents include glycerol (Gly), ethylene glycol (EG), and dimethyl sulfoxide (DMSO) [114, 116]. These solvents are characterized by low freezing points, high polarity, thermal stability, and good thermal conductivity, making them widely suitable for low-temperature applications [102].

**Fig. 5:**
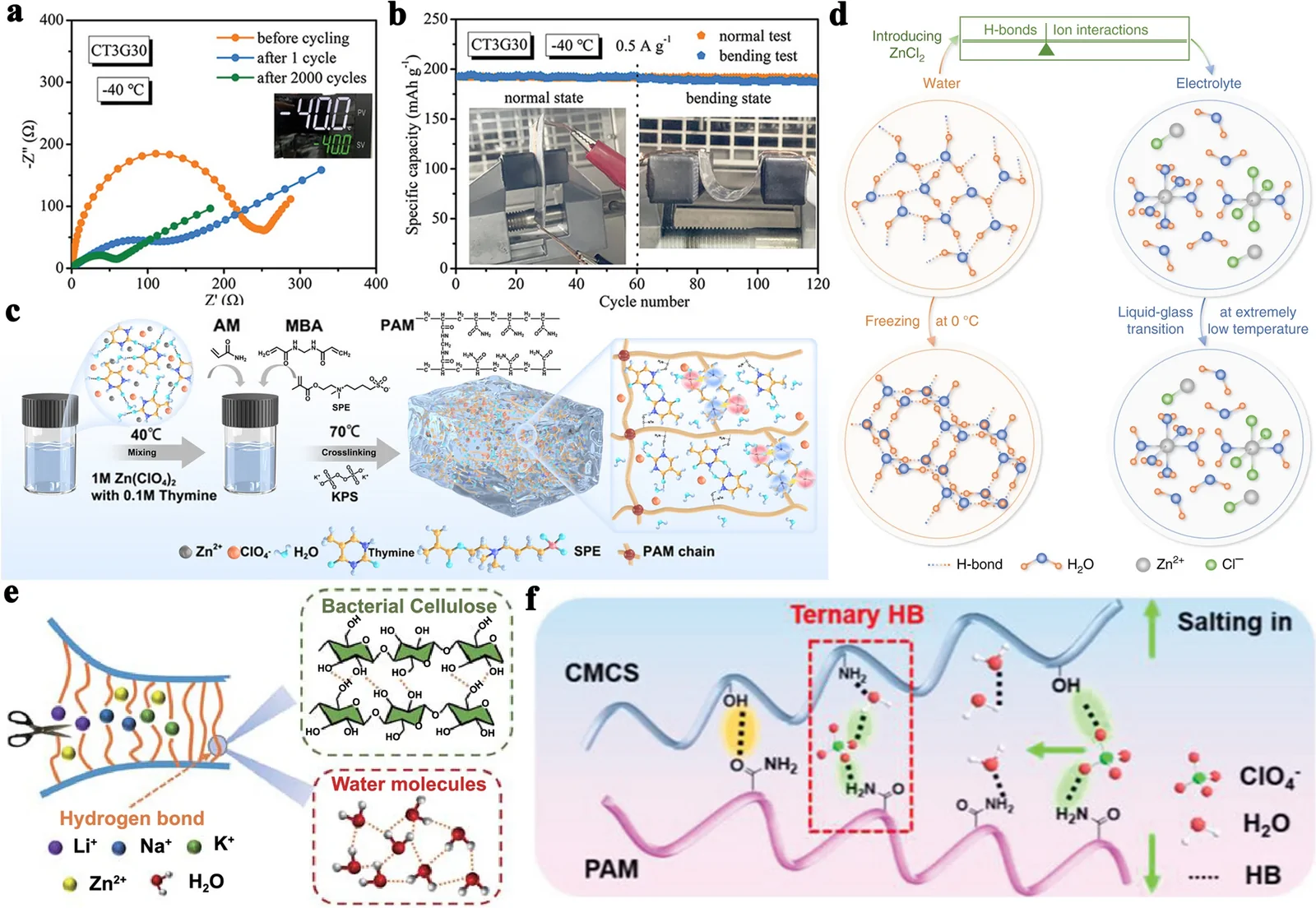
**a** Nyquist plots of ZIBs before cycling, after 1 cycle, and 2,000 cycles at − 40 °C (frequency range 100 kHz–0.01 Hz) and **b** Capacity evolution of the ZIBs with hydrogel before cycling at − 40 °C (0.5 A g^−1^). Reproduced with permission [117]. Copyright 2021, Wiley. **c** Schematic illustration of the fabrication process of the PAM-T-S hydrogel electrolyte. Reproduced with permission [118]. Copyright 2025, Wiley. **d** Schematic of the structure evolutions of water and electrolyte, and the design of low-Tt solution. Reproduced with permission [119]. Copyright 2020, Springer Nature. **e** Schematic illustration of cation-induced disruption of hydrogen bonds between cellulose chains and between water molecules. Reproduced with permission [120]. Copyright 2022, Springer Nature. **f** Schematic illustration of the interactions among carboxymethyl chitosan (CMCS), PAM chains, and ClO_4_^−^ in CSAM-C hydrogel. Reproduced with permission [115]. Copyright 2022, Wiley

For instance, Wong et al. employed glycerol as an anti-freezing agent and integrated it with cellulose using tetraethyl orthosilicate (TEOS) to construct a hydrogel capable of operating at temperatures as low as − 40 °C [117]. The anti-freezing performance stemmed from strong hydrogen bonding interactions between the –OH groups in glycerol and water molecules, which suppressed ice nucleation and preserved flexibility at low temperatures. The presence of ZnSO_4_ and MnSO_4_ further lowered the freezing point, while abundant –OH groups in the cellulose backbone synergistically contributed to ice suppression. This multi-component system enabled the hydrogel to retain both mechanical flexibility and ionic conductivity under extreme cold (Fig. 5a, b).

In another example, Zhou et al. introduced EG into a composite hydrogel based on natural polymers, guar gum (GG) and SA, to create a ternary GG/SA/EG anti-freezing hydrogel electrolyte [121]. The –COO^−^ groups in SA enhanced water retention, while EG formed stable hydrogen bond clusters with water molecules, disrupting the native hydrogen bonding network and effectively lowering the freezing point. Zeng et al. further developed a PAM-T-S hydrogel electrolyte based on a three-component synergistic regulation strategy [118]. This electrolyte incorporated thymine (Thy) and a zwitterionic monomer, [2-(methacryloyloxy)ethyl]dimethyl(3-sulfopropyl)ammonium betaine (SPE), into a polyacrylamide (PAM) matrix to construct a multidimensional cross-linked network (Fig. 5c). Thy formed strong hydrogen bonds with water molecules, restricting free water mobility and significantly delaying ice crystallization. Meanwhile, sulfonate groups in SPE provided rapid Zn^2+^ transport pathways and worked synergistically with Thy to modulate the Zn^2+^ solvation structure, promoting the formation of a stable organic–inorganic composite SEI layer that suppressed side reactions and dendrite growth. Benefiting from this synergy, the hydrogel demonstrated outstanding low-temperature performance, achieving stable cycling for over 3,000 h at − 20 °C.

In summary, the incorporation of organic solvents enables the formation of stable hydrogen bonding networks at the molecular level, effectively regulating the crystallization behavior of water and the solvation environment of Zn^2+^. This strategy offers a practical and flexible approach for endowing aqueous hydrogel electrolytes with robust anti-freezing capabilities. Future research may focus on further improving low-temperature ionic conductivity while maintaining mechanical flexibility, environmental sustainability, and long-term system stability.

In addition to the incorporation of organic solvents, regulating the state of free water in hydrogels through the introduction of salt solutions, such as CaCl_2_, LiCl, ZnCl_2_, NaCl, KCl, Zn(ClO_4_)_2_, and Zn(CF_3_SO_3_)_2_, represents another effective strategy for achieving anti-freezing properties [122]. The core mechanism underlying high-concentration salt-based antifreeze electrolytes lies in modulating ion–water interactions to disrupt the hydrogen bonding network of water. This process lowers the freezing point, suppresses ice crystal formation, and maintains ion transport capability at subzero temperatures [25].

However, excessive salt concentrations can significantly increase the viscosity of the electrolyte and promote salt precipitation as the temperature decreases. To address these limitations, Chen et al. selected highly soluble ZnCl_2_ and optimized its concentration to balance hydrogen bond disruption with controlled ion–solvent interactions with high solubility [119]. By adjusting the ZnCl_2_ concentration to 7.5 M, they successfully reduced the liquid–solid transition temperature of the hydrogel to − 114 °C (Fig. 5d). At this concentration, the proportion of strongly hydrogen-bonded water molecules was substantially reduced, and the electrolyte predominantly contained solvated species such as Zn(H_2_O)_2_Cl_4_^2−^, ZnCl^+^, and Zn(H_2_O)_6_^2+^, effectively inhibiting ice crystal formation. Meanwhile, moderated ion–ion interactions prevented excessive viscosity and precipitation, allowing the hydrogel to maintain its liquid state and good ionic conductivity even at − 70 °C.

Building upon this approach, Xu et al., leveraged the Hofmeister effect and co-introduced ZnCl_2_ and LiCl into the hydrogel electrolyte [120]. Benefiting from the superior hydrogen bond breaking ability of Li^+^, the composite electrolyte more effectively disrupted the hydrogen bonding network among water molecules and the polymer matrix, thereby enhancing ion mobility and broadening the ESW at low temperatures (Fig. 5e). Further advancing the application of the Hofmeister effect, Wu et al. introduced ClO_4_^−^ ions, chaotropic anions known for their ability to weaken hydrogen bonding networks, into the hydrogel electrolyte [115]. Through the formation of weak ternary hydrogen bonds between ClO_4_^−^, water molecules, and polymer chains, the strong inter-water hydrogen bond network was effectively disrupted. This strategy significantly reduced the freezing point of the hydrogel while simultaneously enhancing its flexibility and ionic conductivity at subzero temperatures (Fig. 5f). Unlike conventional methods that rely solely on high salt concentrations, the use of chaotropic ions offers several advantages: It reduces the required salt content, mitigates issues related to viscosity and salt precipitation, and improves the mechanical integrity and interfacial stability of the gel electrolyte.

Overall, the salt-solution-based strategy offers a viable and tunable alternative to organic solvents-based electrolytes for achieving anti-freezing performance in hydrogel electrolytes. By tailoring ion–water interactions, this approach enables the disruption of hydrogen bonding networks, ensuring low-temperature conductivity, structural stability, and reliable interfacial performance. These findings collectively demonstrate the effectiveness of both organic solvent and high-concentration salt strategies in enhancing the low-temperature adaptability of hydrogel electrolytes. A comparative summary of key anti-freezing mechanisms and representative systems is presented in Table 3.

**Table 3:** Summary of recently reported anti-freezing hydrogel electrolytes for ZIBs, including representative material, operating temperature range, and key performance characteristics

Hydrogel substrate	Approach	Freezing tolerance (°C)	Conductivity (mS cm^−1^, °C)	Zn||Zn cell cycle performance	Mechanical properties	Refs
Cellulose	Gly	− 40	19.4, − 40	800 h, 2 mA cm^−2^, − 40 °C	Tensile strength of 2.11 MPa	[117]
PAM	Gly	− 40	0.0965, − 40	–	Strain tolerance of 2760%	[123]
PVA/PMIA	Gly	− 20	9.7, − 20	1,800 h, 1 mA cm^−2^, RT	Tensile strength of 7.8 MPa	[124]
PAM/xanthan gum	Gly	− 20	3.6, − 20	400 h, 0.1 mA cm^−2^, RT	Compressive strength of 0.04 MPa	[125]
GG/SA	EG	− 20	6.19, − 20	200 h, 0.2 mA cm^−2^, − 20 °C	–	[121]
PVA/CMC	EG	− 20	7.5, − 20	700 h, 1 mA cm^−2^, RT	Tensile strength of 0.51 MPa	[126]
PVA/CNF	EG	− 20	42, − 20	500 h, 5 mA cm^−2^, RT	Tensile strength of 6 MPa	[127]
PSBMA/PAM	EG	− 20	8.3, − 20	400 h, 1 mA cm^−2^, − 20 °C	Tensile strength of 0.15 MPa	[128]
PAM	Thy/SPE	− 20	25.3, − 20	3,000 h, 1 mA cm^−2^, − 20 °C	Tensile strength of 0.014 MPa	[118]
PAM/CNF	DMSO	− 40	1.52, − 30	1300 h, 2 mA cm^−2^, RT	Tensile strength of 0.055 MPa	[129]
PVA	DMSO	− 20	–	–	Tensile strength of 0.27 MPa	[130]
Bacterial cellulose	Concentrated salt	− 50	1.14, − 50	700 h, 0.2 mA cm^−2^, − 50 °C	–	[120]
CMCS/PAM	Concentrated salt	− 30	10.4, − 30	1,200 h, 0.5 mA cm^−2^, − 30 °C	Elongation is 320% at − 30 °C	[115]
PANa	Concentrated salt	− 50	5.7, − 20	–	Elongation is 900% at − 50 °C	[131]
Cellulose	Concentrated salt	− 20	74.9, RT	–	Tensile strength of 0.37 MPa	[132]
PAM	Concentrated salt	− 70	2.38, − 70	300 h, 0.5 mA cm^−2^, RT	–	[133]
PAAm	Concentrated salt	− 20	–	–	Tensile strength of 0.064 MPa	[134]
PAM	Concentrated salt	− 20	9.93, − 20	–	Tensile strength of 0.12 MPa	[135]
PSBMA/PAM	Concentrated salt	− 20	10.38, − 20	500 h, 1 mA cm^−2^, − 20 °C	Tensile strength of 0.133 MPa at − 20 °C	[136]

Room temperature (RT)

### High-Temperature Tolerance

As ambient temperature increases, the ionic conductivity of ZIBs generally improves, and electrochemical reaction kinetics are correspondingly accelerated, which can enhance overall battery performance to some extent. However, under elevated temperatures, ZIBs often encounter critical challenges related to the phase stability and interfacial compatibility of hydrogel electrolyte [137]. For instance, when the operating temperature exceeds 45 °C, the HER at the electrolyte–electrode interface becomes more pronounced. This intensification not only accelerates the corrosion and oxidation of electrode materials but also leads to rapid capacity degradation and a shortened battery lifespan. In addition, elevated temperatures exacerbate water evaporation within the gel, increasing the risk of side reactions, internal pressure buildup, and associated safety hazards.

To address these high-temperature issues, researchers have drawn inspiration from anti-freezing electrolyte strategies and proposed the incorporation of high-concentration salts and organic additives to enhance thermal stability and safety under elevated temperature conditions.

Previously, salt solutions have been effectively utilized in anti-freezing hydrogel designs, where increasing salt concentration disrupts the hydrogen bond network of water and suppresses ice crystallization. Under high-temperature conditions, salt-based strategies serve a complementary role: High ion concentrations weaken hydrogen bonding among water molecules, thereby reducing water evaporation, mitigating gel dehydration and shrinkage, and preserving electrolyte structural integrity.

**Fig. 6:**
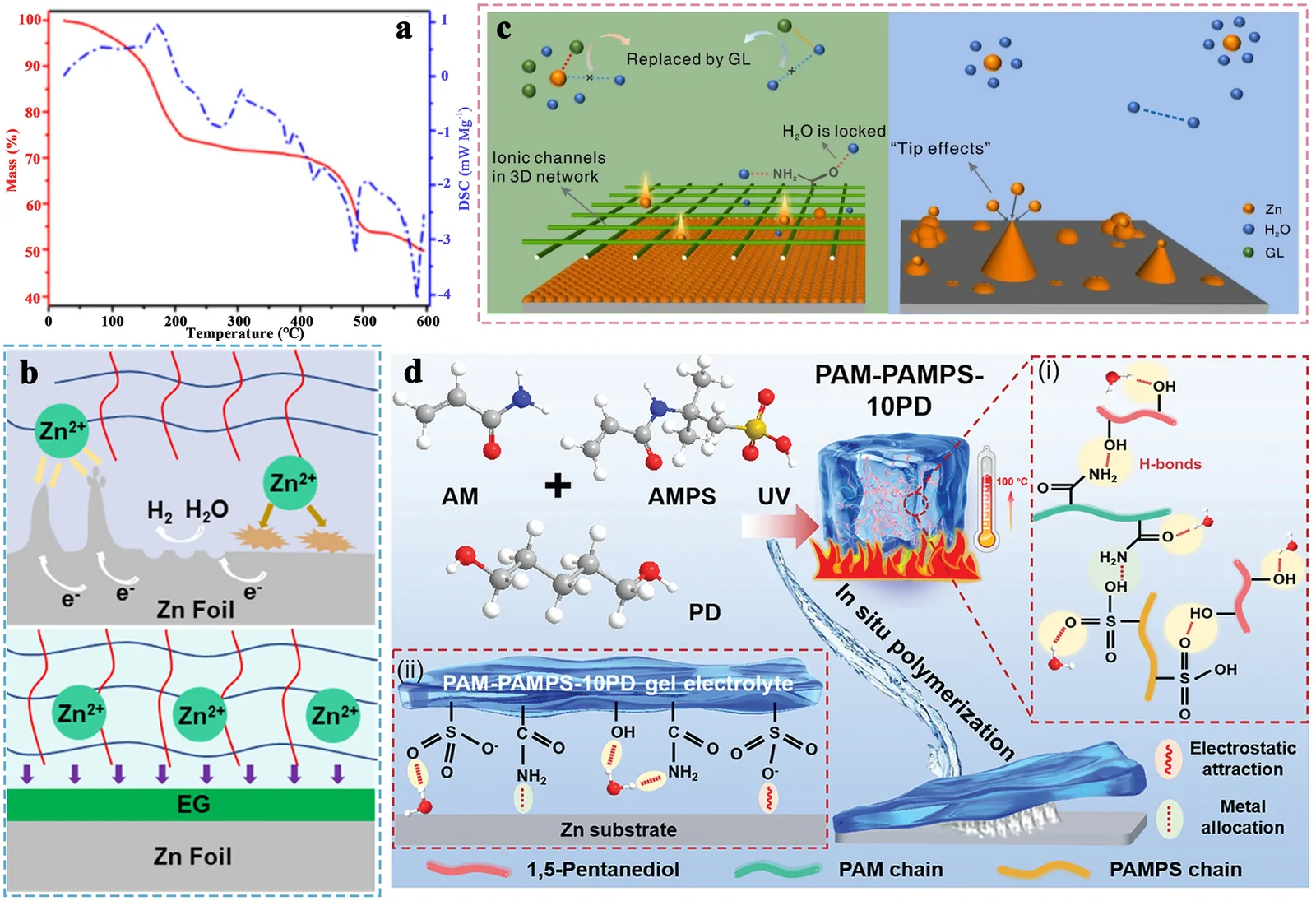
**a** STA characterization of the OSGE electrolyte over the temperature range of RT to 600 °C. Reproduced with permission [138]. Copyright 2021, Elsevier. **b** Schematic diagram showing Zn plating mechanisms when using HE and OHE electrolytes. Reproduced with permission [139]. Copyright 2021, American Chemical Society. **c** Schematic illustration of the possible Zn deposition mechanism in ZnSO_4_/GL/AN hydrogel electrolyte compared with aqueous ZnSO_4_ electrolyte. Reproduced with permission [140]. Copyright 2022, Elsevier. **d** Illustration of the structural design concept for a dual-network polyanionic PAM/PAMPS-10PD electrolyte. Reproduced with permission [141]. Copyright 2025, Wiley

For instance, Tao et al. developed an oversaturated gel electrolyte (OSGE) based on a high-concentration acetate salt [138]. Simultaneous thermal analysis (STA) revealed that OSGE exhibited minimal water loss, only ~ 5% at 100 °C, demonstrating excellent thermal stability and water retention (Fig. 6a). Molecular dynamics simulations further confirmed that Zn^2+^ formed contact ion pairs (CIPs) with CH3COO^−^, which suppressed dendrite formation during Zn deposition and enhanced anode stability. This electrolyte maintained a wide ESW of 3.3 V at 80 °C and achieved 90.6% capacity retention after 300 cycles at 60 °C, demonstrating excellent thermal and electrochemical resilience.

In another study, Li et al. introduced 3 M Zn(CF_3_SO_3_)_2_ into a PAM-based hydrogel to develop a concentrated gel electrolyte for high-temperature ZIBs [142]. The high salt concentration effectively reduced water activity by weakening the hydrogen bonding network among water molecules, significantly enhancing the thermal stability of the system. At 80 °C, this electrolyte delivered a high specific capacity of 501 mAh g^−1^ along with excellent cycling stability. Thermogravimetric analysis (TGA) and X-ray photoelectron spectroscopy (XPS) further indicated that water molecules reversibly intercalated into the cathode interlayers during charging, serving as structural “pillars” to stabilize the electrode. This mechanism improved both the thermal stability and long-term cycling performance of the battery.

These findings collectively highlight that high-concentration salt systems not only regulate water activity but also reinforce the structural integrity of electrode–electrolyte interfaces under thermal stress, providing a viable strategy for enhancing the high-temperature performance of hydrogel electrolytes in ZIBs.

In addition to employing salt solutions to enhance the high-temperature tolerance of hydrogel electrolytes, the incorporation of certain organic solvents has also proven effective in suppressing water evaporation within the hydrogel matrix. These solvents form strong hydrogen bonds with water molecules, thereby reducing water activity, stabilizing the hydrogel structure, and mitigating thermally induced degradation. For example, Xu et al. introduced dimethyl sulfoxide (DMSO) into a polyacrylamide (PAAm) cross-linked hydrogel matrix to construct a multi-component PDZ-H hydrogel electrolyte [143]. DMSO forms strong hydrogen bonds with water, which not only decreases the rate of water evaporation and enhances thermal stability but also suppresses surface passivation on the Zn anode. This dual effect facilitates efficient Zn plating/stripping and improves cycling performance at elevated temperatures.

Ethylene glycol (EG), known for its high boiling point and low vapor pressure, has also been widely used to enhance thermal resilience. Liu et al. developed an EG-based organic hydrogel electrolyte (OHE), in which EG formed hydrogen bonds with water molecules to expand the operating temperature window of the electrolyte [139]. The OHE enabled stable cycling for over 1,000 cycles at 80 °C. Additionally, EG adsorbed onto the Zn anode surface, forming a protective layer that suppressed side reactions, delayed corrosion, and promoted uniform Zn^2+^ deposition (Fig. 6b). Moreover, synergistic effects can be further realized through the rational design of polyol-based composite solvent systems. Hu et al. developed a glycerol (GL)–acetonitrile (AN) hybrid electrolyte, where GL formed hydrogen bonds with water molecules to significantly enhance thermal stability [140]. Meanwhile, AN contributed high ionic conductivity due to its excellent wettability and low viscosity. The synergy between GL and AN enabled stable operation under extreme temperature conditions. Furthermore, the three-dimensional ion transport network in the hybrid system provided abundant conduction pathways, while coordination between GL and Zn^2+^ helped regulate Zn deposition and suppress dendrite formation, ultimately enhancing long-term cycling stability (Fig. 6c).

In another example, Hou et al. incorporated the molecular crowding agent 1,5-pentanediol (PD) into a polyanionic hydrogel system (PAM-PAMPS-10PD) to construct a thermally stable electrolyte capable of operating at 100 °C [141]. PD, as a high-boiling-point organic solvent, formed strong hydrogen bonds with water, effectively suppressing water evaporation and enhancing the thermal stability. In addition, PD modulated the solvation structure of Zn^2+^, reducing anode corrosion and dendrite formation (Fig. 6d). As a result, the PAM-PAMPS-10PD electrolyte maintained stable cycling for over 500 h at 100 °C and achieved a capacity retention of 47.8% after 3,000 cycles in a Zn-AC full battery.

These studies collectively demonstrate that the rational integration of high-boiling-point organic solvents into hydrogel electrolytes offers a powerful strategy to enhance thermal stability. By modulating hydrogen bonding, controlling water activity, and regulating Zn^2+^ solvation, these systems enable safe and stable operation under harsh thermal conditions. A summary of representative high-temperature strategies and their electrochemical performance is presented in Table 4.

**Table 4:** Summary of recently reported high-temperature (high-T) tolerance hydrogel electrolytes

Hydrogel substrate	Approach	High-T tolerance (°C)	Conductivity (mS cm^−1^, RT)	Zn||Zn cell cycle performance	Mechanical properties	Refs
PAA	Concentrated salt	80	3.74, RT	600 h, 0.5 mA cm^−2^, RT	–	[138]
PAM	Concentrated salt	80	27.1, RT	–	–	[142]
PVA	Concentrated salt	80	–	108 h, 0.5 mA cm^−2^, RT	–	[144]
PAM	Concentrated salt	50	15.02, RT	2,000 h, 3 mA cm^−2^, RT	–	[145]
PI/PTC	Concentrated salt	60	6.49, RT	1200 h, 5 mA cm^−2^, RT	–	[89]
PAAm	DMSO	60	41, RT	1,350 h, 2 mA cm^−2^, RT	Tensile strength of 0.048 MPa	[143]
PVA	DMSO	50	30.24, RT	–	Tensile strength of 0.31 MPa	[146]
PAMPS/PAAm	EG	80	21, RT	300 h, 0.2 mA cm^−2^, RT	Tensile strength of 0.18 MPa	[138]
PAM	Gly	60	13.94, RT	500 h, 0.2 mA cm^−2^, 60 °C	–	[139]
PAM	1,5-Pentanediol	100	63.97, RT	500 h, 0.5 mA cm^−2^, 100 °C	Tensile strength of 0.006 MPa	[140]

Poly(vinylidenefluoride-co-trifluoroethylene-co-chlorotrifluoroethylene) (PTC), Polyimide (PI), Room temperature (RT)

### Thermoresponsive Properties

As previously discussed, high-temperature environments pose serious challenges to the stability and safety of ZIBs. In addition to improving thermal tolerance through component regulation, recent advances have led to the development of hydrogel electrolytes with intrinsic high-temperature self-protection capabilities. These thermoresponsive electrolytes offer novel strategies for the safe operation of flexible ZIBs under extreme conditions [147]. These electrolytes exhibit thermal-responsive behavior: when the temperature exceeds a predetermined threshold, the hydrogel undergoes a structural reconfiguration or phase transition that interrupts ion transport between the electrolyte and electrodes, thereby achieving a self-terminating protective function. Once the temperature returns to a safe range, some of these materials are capable of self-healing, reestablishing the ion conduction network and restoring normal battery operation. Such intelligent thermoresponsive electrolytes provide an essential safety mechanism for ZIB thermal management and hold great promise for practical applications. Currently, two main approaches have been adopted to realize thermally responsive behavior in hydrogel electrolytes: (1) thermosensitive sol–gel systems that undergo reversible phase transitions and (2) hygroscopic hydrogel systems with dual responsiveness to temperature and humidity.

**Fig. 7:**
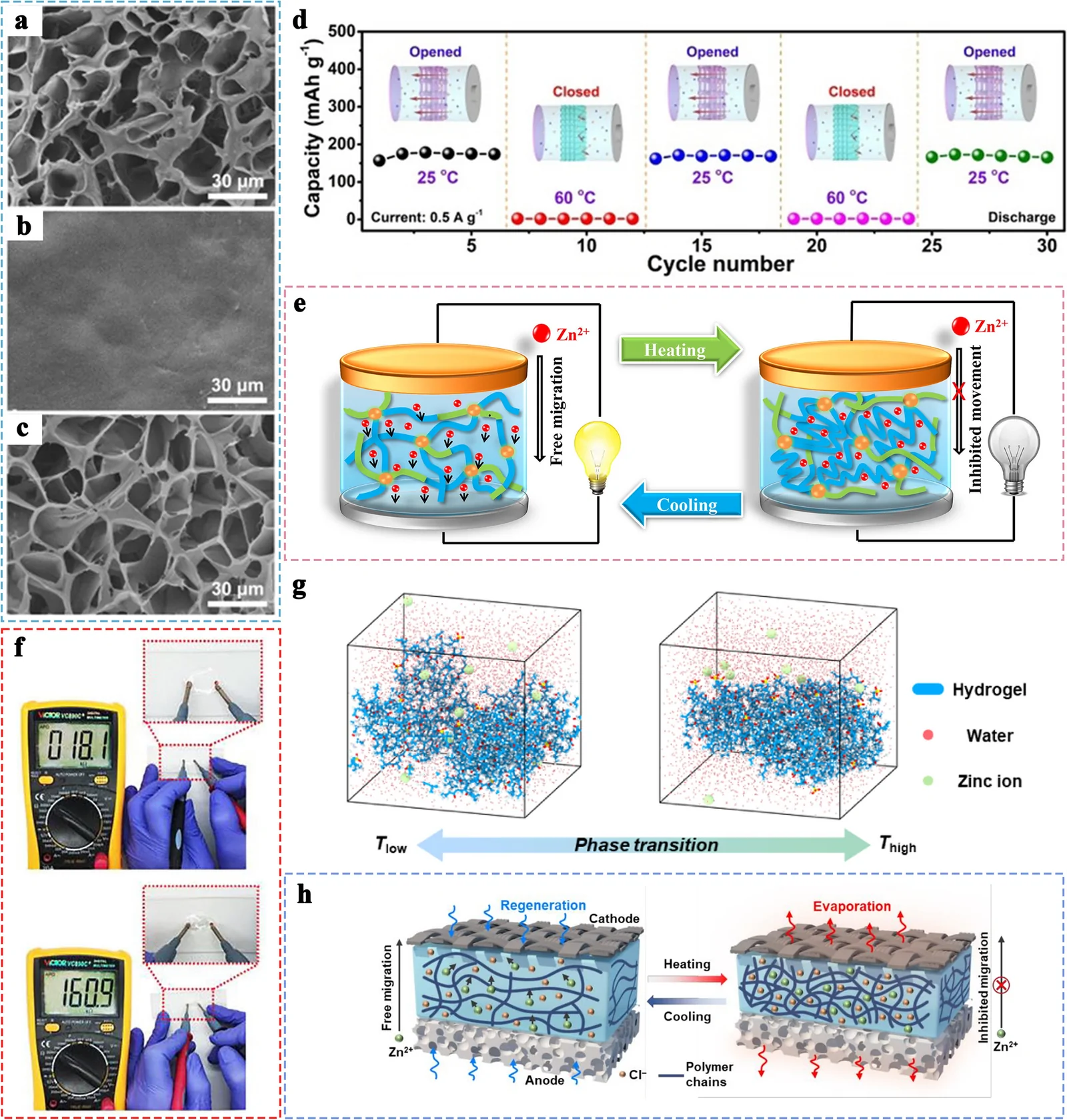
SEM images of PNIPAM/AM-5@GF separators at different thermal states: **a** 25 °C, **b** 60 °C, and **c** after cooling to 25 °C. **d** Thermal-responsive reversibility of aqueous ZIBs with thermal-gated separators evaluated at 25 °C and 60 °C over multiple cycling stages (0.5 A g^−1^). Reproduced with permission [149]. Copyright 2020, Wiley. **e** Schematic of a thermoresponsive sol–gel system enabling on-demand phase switching in a ZIB. **f** Comparison of the electrical conductivity of PNA in its sol state and gel state. Reproduced with permission [66]. Copyright 2018, Elsevier. **g** Snapshots showing the thermal-induced phase transition process, before (left) and after (right). Reproduced with permission [150]. Copyright 2024, Elsevier. **h** Working mechanism of the thermal self-protective ZIBs based on hygroscopic hydrogel electrolyte. Reproduced with permission [151]. Copyright 2020, Wiley

Hydrogels based on poly(N-isopropylacrylamide) (PNIPAM) exhibit typical reversible sol–gel phase transition behavior. Above its lower critical solution temperature (LCST, ~ 32 °C), PNIPAM undergoes a coil-to-globule transition, causing the three-dimensional polymer network to shrink due to hydrophobic interactions [148]. When the temperature falls below the LCST, the structure re-expands, exhibiting excellent reversibility and structural memory. Building on this concept, Niu et al. developed a composite hydrogel by copolymerizing NIPAM with acrylamide (AM) to form a PNIPAM/AM network [149]. The incorporation of hydrophilic AM groups enhanced hydrogen bonding with water molecules, thereby increasing the LCST to 45 °C, better aligning with typical ZIB operating conditions. At room temperature, the hydrogel exhibited a porous structure favorable for electrolyte uptake. Above the LCST, the network contracted, sealing the ion conduction channels. Upon cooling, the network reopened, enabling reversible thermal switching of ionic conductivity (Fig. 7a–c). Temperature-cycling tests further confirmed the hydrogel’s ability to repeatedly block ion transport at 60 °C and recover its full electrochemical performance upon cooling (Fig. 7d).

To improve temperature adaptability, Zhi et al. developed a reversible sol–gel electrolyte based on poly(N-isopropylacrylamide-co-acrylic acid) (PNA) [66]. At room temperature, the electrolyte remained in a low-viscosity sol state, facilitating Zn^2+^ transport. Upon heating, the system underwent phase separation and physically cross-linked into a gel due to hydrophobic interactions, sharply increasing ionic resistance (Fig. 7e). By tuning the NIPAM-to-AA molar ratio, the transition temperature could be precisely adjusted. Electrochemical impedance spectroscopy showed that the electrolyte’s resistance surged from 18.1 to 160.9 MΩ at 70 °C (Fig. 7f), effectively interrupting the battery reaction pathway and achieving temperature-triggered self-protection.

Addressing both dendrite suppression and thermal safety, Yang et al. synthesized a dual-functional hydrogel by copolymerizing PNIPAM with 2-acrylamido-2-methylpropanesulfonic acid (AMPS) [150]. The sulfonic acid groups in AMPS, possessing strong Zn^2+^ affinity, guided uniform ion distribution and reduced electric field gradients, thereby inhibiting dendrite growth. The NIPAM/AMPS composition raised the LCST to 80 °C. Above this threshold, hydrophobic chain contraction reduced ionic conductivity by over 90%, providing automatic shutdown at elevated temperatures (Fig. 7g). The symmetrical cell with the hydrogel maintained over 1,000 h of stable cycling at room temperature while offering built-in thermal protection.

Despite these advances, current thermoresponsive hydrogels are mostly based on PNIPAM, which presents limitations such as potential cytotoxicity and the need for high polymer concentrations (> 20 wt%) to ensure gel formation, which in turn compromises ionic conductivity. To overcome this, Jiang et al. developed a methylated chitin (MCH)-based hydrogel with a tunable gelation temperature range (15–85 °C) at only 3 wt% polymer content [152]. When integrated into ZIBs, the MCH electrolyte rapidly gelled in response to thermal runaway, sharply increasing internal resistance and effectively disconnecting the electrochemical circuit, achieving fast and efficient self-protection.

In contrast to sol–gel phase transition systems, hygroscopic hydrogels offer dual responsiveness to temperature and humidity. Fan et al. designed an intelligent hydrogel utilizing ZnCl_2_’s deliquescent properties to regulate internal moisture content for thermally responsive protection (Fig. 7h) [151]. When heated to 50 °C, water evaporated rapidly through porous electrodes, cooling the system and reducing surface temperature by 12.5 °C. At the same time, water loss drastically decreased the Zn^2+^ diffusion coefficient from 3.8 × 10^−10^ to 3.4 × 10^−11^ cm^2^ s^−1^, effectively halting electrochemical reactions. Upon cooling, the hydrogel reabsorbed ambient moisture and restored ionic conductivity, enabling reversible, humidity-driven thermal protection.

In summary, thermoresponsive hydrogel electrolytes offer unique advantages for ensuring the operational safety of ZIBs in high-temperature environments. Two principal design strategies have emerged. On one hand, reversible sol–gel systems based on thermosensitive polymers (e.g., PNIPAM), which respond to heat by undergoing a phase transition that increases ion transport resistance and enables automatic shutdown. Upon cooling, these systems reestablish their conductive networks, exhibiting excellent reversibility and thermal memory. On the other hand, hygroscopic hydrogels based on moisture-sensitive salts (e.g., ZnCl_2_), which exploit water evaporation and reabsorption to achieve temperature-triggered control over ionic conductivity. Together, these two strategies not only enhance the thermal stability of ZIBs but also provide crucial design insights for developing safe, flexible, and adaptive energy storage devices operable under extreme environmental conditions.

### Self-Healing Performance

Flexible energy storage devices are particularly susceptible to structural damage or interfacial delamination of hydrogel electrolytes after prolonged cycling or repeated mechanical deformation, which can significantly reduce energy efficiency and even lead to device failure [153]. Self-healing hydrogel electrolytes can autonomously restore their microstructure and electrochemical function by reestablishing reversible bonds at damaged sites, thereby maintaining structural integrity and prolonging battery lifespan [154, 155]. The key to these systems lies in their intrinsic self-repairing capabilities, which are enabled by dynamic chemical or physical cross-linking interactions. Based on the underlying healing mechanisms, self-healing hydrogels can be broadly categorized into physically cross-linked and chemically cross-linked systems [156].

**Fig. 8:**
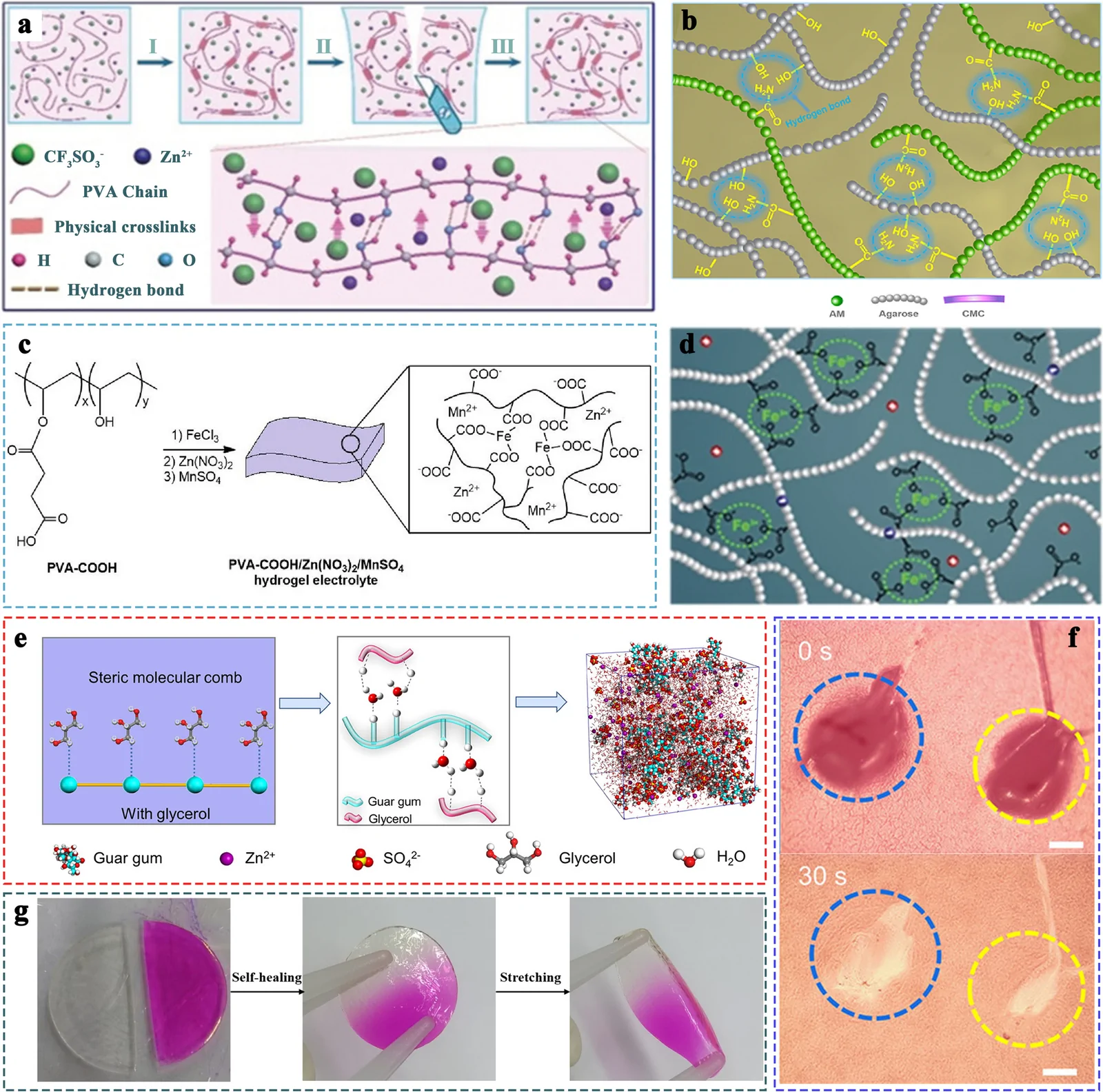
**a** Process of fabricating the self-healing hydrogel electrolyte and demonstration of its recovery behavior: (I) freezing (15 h, − 18 °C) and thawing (24 h, room temperature) to form the 3D polymer network, (II) cutting to introduce mechanical damage, and (III) subsequent self-healing at room temperature within minutes, demonstrating effective reconnection and restoration of mechanical integrity. Reproduced with permission [159]. Copyright 2019, Wiley. **b** Schematic illustration of the origin of self-healability arising from hydrogen bond interactions. Reproduced with permission [61]. Copyright 2021, Elsevier. **c** Schematic illustration of the preparation process of the self-healable PVA-COOH/Zn(NO_3_)_2_/MnSO_4_ hydrogel electrolyte. Reproduced with permission [160]. Copyright 2019, American Chemical Society. **d** Schematic illustration of the origin of self-healability in hydrogel electrolytes. Reproduced with permission [161]. Copyright 2018, Wiley. **e** Schematic illustration of the steric molecular combing effect in stretching guar gum molecules with glycerol, with a 3D MD snapshot of the guar gum/ZnSO_4_/glycerol system. **f** Optical images showing the rapid self-healing behavior of the glycerol-borate hydrogel electrolyte. Reproduced with permission [162]. Copyright 2022, American Chemical Society. **g** Images highlighting the dynamic self-healing capability of the PAM-PAAS-QCS hydrogel. Reproduced with permission [163]. Copyright 2024, American Chemical Society

Physically cross-linked self-healing hydrogels rely on non-covalent interactions such as hydrogen bonding, metal coordination, and π–π stacking to construct dynamic networks [157]. These interactions can be spontaneously reformed after mechanical damage. Among them, hydrogen bonding networks formed through polar functional groups (e.g., –OH, –COOH) on polymer chains, such as PVA and SA, act as sacrificial cross-links. When subjected to mechanical stress, hydrogen bonds rupture to dissipate energy and subsequently reform upon relaxation, enabling self-repair [158]. For example, Niu et al. designed a PVA/Zn(CF_3_SO_3_)_2_ hydrogel electrolyte via a freeze–thaw strategy to form a porous 3D network [159]. Zn^2+^ ions coordinated with hydroxyl groups on PVA chains, while crystalline domains generated during freezing provided mechanical support. This dual-network architecture offered both structural rigidity and reversible dynamics. The addition of Zn(CF_3_SO_3_)_2_ not only enhanced ionic conductivity (up to 12.6 mS cm^−1^) by optimizing ion pathways but also reduced the crystalline content, improving transparency and chain mobility. Upon mechanical damage (Fig. 8a, step II-III), exposed non-crystalline PVA segments facilitated hydrogen bond reformation, leading to full restoration of mechanical and electrochemical properties, with scratches healing within minutes and ionic conductivity remaining stable even after 10 cutting/healing cycles, while the healed gel could support 100 g without fracture. Huang et al. further developed a self-healing hydrogel composed of agarose, PAM, and CMC, where abundant –OH and –CONH_2_ groups facilitated extensive hydrogen bonding. A rigid-flexible double-network was formed via free-radical polymerization, offering excellent mechanical strength and healing ability (Fig. 8b) [61]. The hydrogel electrolyte delivered an ionic conductivity of 23.1 mS cm^−1^ at room temperature and enabled flexible Zn-MnO_2_ batteries to retain 83.1% capacity after 1500 cycles at 5.0 A g^−1^, while maintaining over 95% healing efficiency after five complete cutting/healing cycles.

Metal coordination is another vital mechanism for physical cross-linking. Polymers bearing functional groups such as –COOH, –OH, and –NH_2_ can reversibly coordinate with metal ions (e.g., Fe^3+^, Cu^2+^, and Zn^2+^), generating dynamic networks with robust mechanical integrity and self-repairability. For example, Pan et al. constructed a carboxyl-modified PVA-Fe^3+^ hydrogel electrolyte by grafting succinic anhydride onto PVA to introduce carboxyl groups [160]. The resulting dynamic COO^–^–Fe^3+^ coordination bonds endowed the hydrogel with high ionic conductivity (10.9–25.8 mS cm^−1^), mechanical strength, and a healing efficiency of up to 99.6% within 10 min after cutting (Fig. 8c). This hydrogel retained stable performance in Zn-MnO_2_ quasi-solid-state batteries even after multiple cut-heal cycles. Similarly, Zhi et al. fabricated a Fe^3+^-coordinated polyacrylate (PANa) hydrogel for NiCo||Zn batteries, where lone pair electrons on oxygen atoms in acrylate groups coordinated reversibly with Fe^3+^ ions to form a dynamic ion-cross-linked network (Fig. 8d) [161]. The healed hydrogel exhibited tensile strain and strength of about 1000% and 205 kPa (vs. 63% and 30 kPa for pure PANa), and the NiCo||Zn battery delivered nearly 250 mAh g^−1^ with 87% capacity retention after four healing cycles, demonstrating reliable electrochemical and self-healing performance.

Chemically cross-linked self-healing hydrogels leverage reversible covalent interactions such as imine bonds, disulfide bonds, Diels–Alder adducts, and borate esters. These dynamic covalent bonds enable structural restoration upon damage while contributing to mechanical strength. For instance, Mai et al. introduced borate ions into a guar gum matrix to fabricate a quasi-solid-state electrolyte with ultrafast healing ability [162]. Glycerol was added to induce steric hindrance, thereby exposing hydroxyl groups that formed dynamic borate ester cross-links (Fig. 8e). This hydrogel rapidly repaired puncture sites, caused by zinc dendrites, within ~ 30 s at room temperature without external stimuli (Fig. 8f), far outperforming conventional healing systems in speed and interfacial adaptability.

In another study, Qu et al. developed a dual-network PAM-PAAS-QCS hydrogel [163]. Quaternized chitosan (QCS) chains reacted with glutaraldehyde to form dynamic imine bonds, while the network also benefited from hydrogen bonding and ionic interactions. A dye-labeled fracture experiment showed complete interfacial reconnection and homogeneous dye diffusion after 48 h, confirming its effective self-healing capability (Fig. 8g). Currently, self-healing hydrogel electrolytes are predominantly constructed through dynamic cross-linking mechanisms including hydrogen bonding, metal coordination, imine bonding, and borate ester formation. In general, physically cross-linked hydrogels based on hydrogen bonding or metal coordination often exhibit rapid healing efficiency due to the reversibility of non-covalent interactions, but their mechanical strength is relatively limited. By contrast, chemically cross-linked systems relying on dynamic covalent bonds usually exhibit better mechanical stability, but their healing process is relatively slow and sometimes requires external assistance. To balance these trade-offs, hybrid strategies combining physical and chemical interactions have emerged as promising approaches.

These interactions allow rapid restoration after mechanical damage and contribute to energy dissipation, enhancing mechanical toughness and fatigue resistance. Despite significant progress, challenges remain in balancing mechanical robustness with healing efficiency, as well as maintaining cross-link reversibility under operational stress. Long-term performance under complex deformation and harsh environments remains a key hurdle. It is also worth noting that self-healing behaviors in hydrogel electrolytes based on dynamic covalent bonds are strongly influenced by external stimuli. In such systems, bond cleavage and reformation typically occur under thermodynamic equilibrium, but often require triggers such as pH variation, temperature change, or light irradiation to achieve effective healing. While these stimuli can promote reversible network reconstruction, they also impose restrictions on practical design, as excessive dependence on external control may compromise device simplicity and applicability in ZIBs. Therefore, the future development of self-healing hydrogel electrolytes should emphasize multi-mechanism synergy, rapid and autonomous healing, and broad environmental adaptability, which are crucial for enabling next-generation flexible and durable energy storage systems.

### Other Functionalization Strategies

To further broaden the application scope of hydrogel electrolytes in complex environments and diverse operational scenarios, researchers have explored a variety of advanced functionalization strategies beyond core capabilities such as anti-freezing, high-temperature tolerance, thermal responsiveness, and self-healing. These additional strategies aim to enhance the environmental adaptability, safety, and long-term durability of hydrogel electrolytes through multifunctional design incorporating flame retardancy, anti-aging, biocompatibility, and sustainability.

**Fig. 9:**
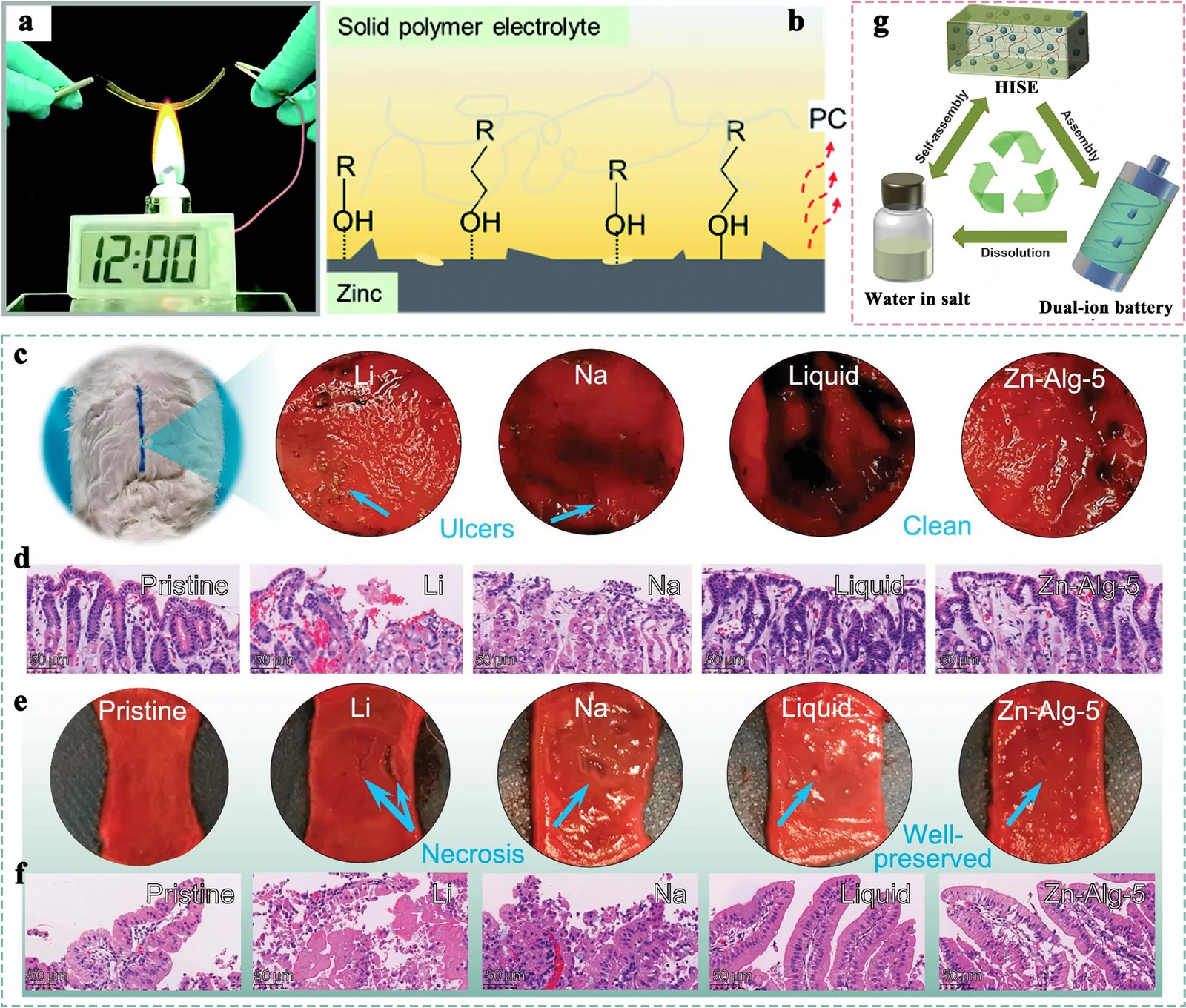
**a** Photographic evidence of a battery ignited by the flame of an alcohol lamp, evidencing its superior flame-retardant property. Reproduced with permission [164]. Copyright 2020, The Royal Society of Chemistry. **b** Schematic diagram highlighting surface interactions and intermolecular forces within ABSPEs. Reproduced with permission [165]. Copyright 2020, The Royal Society of Chemistry. **c-f** Schematic illustration of implantable ZIBs tested in rabbits, demonstrating stable in vivo performance and excellent biocompatibility without detectable inflammation during the 14-day implantation test. Reproduced with permission [166]. Copyright 2022, Oxford University Press. **g** Illustration of a recyclable hydrogel system, highlighting its structural features and regeneration process. Reproduced with permission [167]. Copyright 2022, Elsevier

For instance, to mitigate the risk of thermal runaway under high energy density operation, flame-retardant additives, such as SiO_2_ nanoparticles and phosphorus-containing compounds, have been introduced to improve the fire resistance of gel electrolytes. Huang et al. developed an ionogel electrolyte doped with SiO_2_ nanoparticles and applied it to Zn//MnO_2_ wire-shaped flexible batteries [164]. The incorporation of SiO_2_ significantly improved both flame retardancy and system flexibility. Upon exposure to open flame, SiO_2_ particles migrated to the polymer surface and formed a compact silica–carbon barrier layer, effectively isolating heat and oxygen and thereby suppressing combustion. Remarkably, the Zn//MnO_2_ battery maintained normal operation of an electronic clock even during direct flame exposure, highlighting the excellent fire safety of this system under extreme conditions (Fig. 9a).

Another critical challenge for hydrogel electrolytes is the aging effect, typically characterized by structural densification, water loss, and increasing interfacial resistance, ultimately leading to performance degradation over time. To address this, Parkin et al. developed an anti-aging bonding solid polymer electrolyte (ABSPE) for application in rechargeable ZIBs [165]. The electrolyte employed poly(ethylene glycol) diglycidyl ether (PEGDGE) as the polymer matrix, Zn(OTf)_2_ as the zinc salt, and propylene carbonate (PC) as a plasticizer to form a robust three-dimensional cross-linked network. This architecture facilitated the formation of hydrogen bonds and Zn–O–C coordination interactions at the electrode–electrolyte interface, thereby enhancing interfacial adhesion and suppressing the growth of interfacial resistance (Fig. 9b). Long-term aging tests demonstrated that the interfacial resistance of ABSPE remained nearly unchanged over 200 h, significantly outperforming conventional hydrogel electrolytes and showcasing its superior interfacial stability and anti-aging properties.

With the increasing proliferation of wearable and implantable electronic devices, the biocompatibility of electrolyte materials has emerged as a critical research focus. Traditional energy storage systems often suffer from limitations such as the cytotoxicity of organic solvents or transition metal salts, as well as insufficient mechanical compliance, rendering them unsuitable for applications in biological environments where safety and flexibility are paramount. In contrast, bio-friendly hydrogel-based electrolytes offer unique advantages in terms of softness, conformability, and biological safety. For example, Zhou et al. developed a multilayer Zn-alginate (Zn–Alg) hydrogel electrolyte via a green electrochemical approach [166]. This electrolyte was cross-linked through interactions between carboxylate groups and Zn^2+^ ions, thereby eliminating the need for chemical initiators typically used in polymerization. The resulting Zn–Alg hydrogel demonstrated excellent controllability in synthesis, high biocompatibility, and favorable electrochemical properties. In in vivo experiments involving battery implantation into rabbit gastric and duodenal tissues, histological analysis after 6 h revealed that only the Zn–Alg electrolyte system caused no observable mucosal damage, thereby confirming its safety for biomedical applications (Fig. 9c–f).

While biocompatibility is essential for the safe operation of implantable devices, sustainability represents another crucial dimension when evaluating electrolyte materials across their full life cycle. Increasing efforts have been devoted to developing electrolyte systems that incorporate renewable resources or biodegradable polymers, aiming to minimize environmental impact while maintaining strong electrochemical performance. For instance, Liu et al. proposed a recyclable and biodegradable hydrogel electrolyte composed of polyvinyl alcohol (PVA) and gelatin, combined with a high-concentration ZnCl_2_ salt (Fig. 9g) [167]. This electrolyte exhibited rapid environmental degradation, completely disintegrating in freshwater within 3 h. Cytotoxicity assays confirmed that the diluted degradation products posed negligible biological toxicity. Importantly, even after five recycling cycles, the electrolyte retained nearly constant ionic conductivity, thereby demonstrating its excellent reusability, biocompatibility, and eco-friendliness.

In conclusion, beyond the widely discussed functionalities such as anti-freezing, self-healing, and thermal responsiveness, hydrogel electrolytes also offer promising advantages in terms of flame retardancy, anti-aging, biocompatibility, and sustainability. These extended functionalities not only enhance the resilience and safety of energy storage systems under extreme conditions but also expand their application potential in wearable, implantable, and environmentally friendly energy devices. With their comprehensive performance attributes, hydrogel electrolytes are poised to become a next-generation alternative to conventional liquid electrolytes and are expected to play a pivotal role in the development of high-performance, long-lifespan, and green energy storage technologies. A comparative summary of these functional optimization strategies and their impact on electrolyte performance is presented in Table 5.

**Table 5:** Functional optimization strategies for hydrogel electrolytes and their corresponding effects on electrochemical performance in ZIBs

Approach	Representative materials/strategies	Anti-freezing	High-temperature	Thermo-responsive	Self-healing	Biocompatibility
Organic solvents	Gly, EG, DMSO	√	√	×	△	△
Concentrated salt	ZnCl_2_, LiCl	√	√	×	×	△
Stimuli-responsive polymers	PNIPAM, MCH	×	√	√	×	△
Dynamic cross-linked network	Hydrogen bonding, metal coordination, Imine bonds	×	×	×	√	△
Natural polymer matrix	CMC, Gelatin, Alginate	√	√	×	√	√

*The symbols “√,” “△,” and “ × ” in the table represent significant enhancement, partial enhancement, and no apparent effect of the corresponding optimization strategy on the target function, respectively

## Applications

With the continuous expansion of application scenarios for energy storage devices, the limitations of traditional liquid electrolytes in terms of flexibility, safety, and environmental adaptability have become increasingly apparent, thereby accelerating the development of novel electrolyte materials represented by hydrogels. Meanwhile, ongoing improvements in the material properties of hydrogel electrolytes have further promoted their widespread application in ZIBs, where they demonstrate distinct advantages. Among various application directions, flexible electronic devices and biocompatible electronic devices are particularly representative: The former imposes higher demands on material bendability, fatigue resistance, and operational stability, while the latter emphasizes biosafety, biodegradability, and electrochemical behavior in physiological environments. Owing to their multifunctional advantages, hydrogel electrolytes exhibit strong adaptability and promising prospects in both of these fields.

### Flexible Electronic Devices

**Fig. 10:**
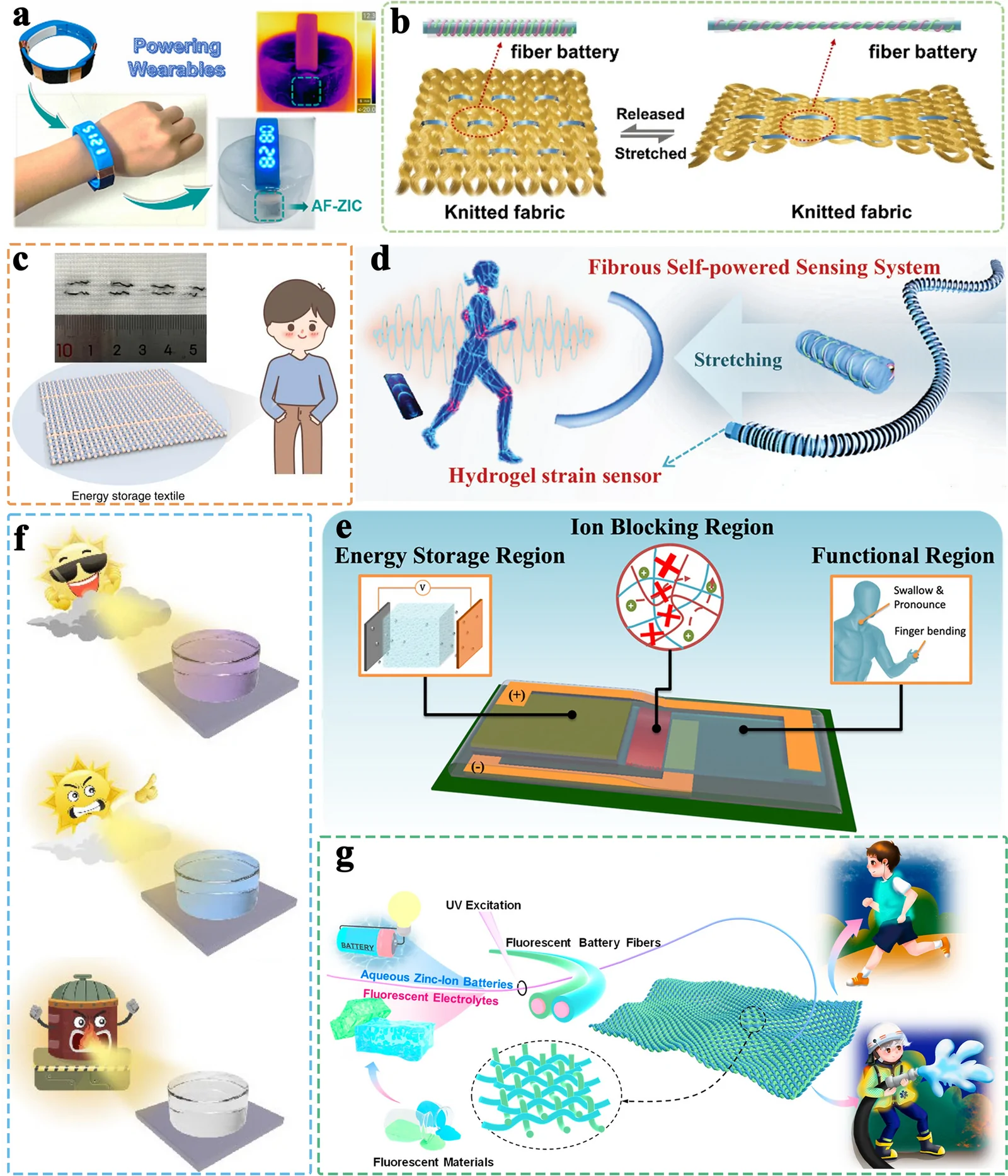
**a** Photographic demonstration of the tandem device powering a wristwatch under practical conditions, including bending on the wrist and encapsulation in ice, confirming stable power delivery and excellent environmental adaptability. Reproduced with permission [168]. Copyright 2023, The Royal Society of Chemistry. **b** Schematic diagram illustrating the integration of stretchable ZIBs with knitted fabric and their mechanical stretching behavior. Reproduced with permission [169]. Copyright 2023, Springer Nature. **c** Photograph showing yarn-shaped ZIBs woven into textiles. Reproduced with permission [170]. Copyright 2024, Elsevier. **d** Schematic representation of the structural integration and functional design of fibrous self-powered sensing systems. Reproduced with permission [171]. Copyright 2024, Wiley. **e** Schematic diagram illustrating the working principle of a self-powered strain sensor. Reproduced with permission [127]. Copyright 2024, American Chemical Society. **f** Schematic illustration of the thermochromic behavior of the hydrogel. Reproduced with permission [172]. Copyright 2022, American Chemical Society. **g** Schematic diagram of textile consisting of ZIBs. Reproduced with permission [173]. Copyright 2023, American Chemical Society

In the context of the growing popularity of flexible electronics, energy storage systems face multiple challenges such as limited space, complex structures, and frequent mechanical deformation. Hydrogel electrolytes, with their excellent flexibility, compressibility, and stress-buffering capacity, serve as ideal candidates for flexible ZIBs. Particularly in wearable devices like smart wristbands and smartwatches, the electrolyte must maintain long-term skin contact and withstand repeated bending, which places higher demands on mechanical durability and electrochemical stability. Yang et al. developed a supramolecular zwitterionic hydrogel electrolyte (SZHE) that not only delivers high ionic conductivity (up to 48 mS cm^–1^) but also demonstrates exceptional stretchability (2150% elongation), self-healing ability, and environmental resilience (stable operation from − 40 to 60 °C) [168]. When three SZHE-based flexible ZIBs were connected in series to power a smartwatch as a strap, the device continued to function even after being frozen in an ice block, confirming SZHE’s practical utility under extreme conditions (Fig. 10a). In addition, the assembled devices exhibited high energy densities of 169.7 Wh kg^–1^, specific capacities of 212.1 mAh g^–1^, and remarkable cycling stability with 88.1%–96.5% capacity retention over 50,000 cycles across − 20 to 60 °C, underscoring its application potential.

Furthermore, flexible ZIBs can serve not only as independent power sources adhered to the device surface, but also be integrated more deeply into wearable platforms such as clothing and accessories through structural design. For example, by weaving hydrogel-based flexible ZIBs into textile substrates, it is possible to construct smart electronic fabrics that combine energy storage functionality with comfortable wearability. Wei et al. developed a stretchable fiber-shaped ZIB using carbon yarn/NiCo_2_S_4-x_ nanotube arrays combined with a PANa hydrogel electrolyte, enabling successful weaving into textile structures (Fig. 10b), and achieving a high capacity of 271.7 mAh g^–1^ with stable cycling and mechanical resilience under stretching and bending [169]. In addition, Jia et al. further proposed a coaxial winding strategy using highly conductive CNTs and high-capacity V_2_O_5_·nH_2_O nanowire composite materials to form core–shell fiber electrodes [170]. A PAM-based hydrogel electrolyte was used to construct fiber-shaped ZIBs with excellent weavability and stable electrochemical performance under mechanical deformation (Fig. 10c), delivering a high capacity of 566.7 mAh g^–1^ at 0.1 A g^–1^ with an energy density of 471 Wh kg^–1^ and retaining 53.1% capacity after 5,000 cycles.

To further extend the application of hydrogel electrolytes in flexible electronics, integration with various sensing modules has become a research hot spot. Compared with using flexible ZIBs solely as power sources, integrating them with sensors to construct “energy storage + sensing” platforms enable real-time monitoring of physiological signals or environmental changes, enhancing the integration and intelligence of wearable systems. Cao et al. designed a dual-functional hydrogel based on MXene-grafted poly(acrylic acid) (MXene-g-PAA), enabling simultaneous strain sensing and energy storage [171]. With a monolithic configuration that integrates ZIB and sensing units, the device not only maintained a high volumetric capacity of 353 mAh cm^–3^ over 400 cycles but also exhibited reliable strain sensitivity (gauge factor of 2.4) within an ultra-wide range of 0–800%, allowing real-time monitoring of body movements such as muscle contraction and motion (Fig. 10d). Moreover, Ma et al. utilized a PVA/nano-SiO_2_/cellulose nanofiber composite hydrogel that served both as the electrolyte for ZIBs and the functional layer for a strain sensor [127]. Without the need for external power, the device can detect body movements such as finger bending, swallowing, and vocalization in real time (Fig. 10e), while maintaining high ionic conductivity (72 mS cm^–1^ at room temperature, 42 mS cm^–1^ at − 20 °C), stable cycling over − 20 to 80 °C with 88% retention after 250 cycles, and reliable strain-sensing performance across 1000 cycles.

In addition to integration with sensing modules, hydrogel electrolytes can respond to external stimuli to enable intelligent perception and feedback. For example, Huang et al. constructed a thermochromic hydrogel electrolyte using PAM, CNF, and CMC, combined with thermochromic capsule powder, to endow fiber-shaped ZIBs with temperature-responsive functionality [172]. The device undergoes a rapid and reversible color change within the 25–60 °C range, enabling visible temperature sensing (Fig. 10f), while delivering a high capacity of 383 mAh g^–1^ at 0.5 A g^–1^ with 98% and 81% retention after 1,000 and 3,500 cycles, respectively. Such responsive hydrogels can be integrated into wearable devices for real-time temperature monitoring and overheating warnings.

In addition to thermal response, hydrogel electrolytes can also provide visual feedback through fluorescence. Zhang et al. introduced fluorescent carbon dots (CDs) into hydrogel electrolytes, enabling fiber-shaped ZIBs to simultaneously offer energy storage and multicolor luminescence under UV light [173]. These ZIB fibers can be woven into textiles to construct intelligent wearable systems with both illumination and energy storage functions, suitable for applications such as nighttime identification and emergency signaling (Fig. 10g), delivering a high volumetric capacity of 92.0 mAh cm^–3^ at 0.1 A cm^–3^, an energy density of 0.17 Wh cm^–3^, and 78.9% capacity retention after 1,500 cycles.

### Biocompatible Electronic Devices

In bio-related energy devices, the biosafety and biodegradability of materials have become key criteria for evaluating their application potential. Owing to their inherent flexibility, hydrophilicity, and tissue-like structure, hydrogel electrolytes exhibit excellent biocompatibility, effectively mitigating inflammatory responses caused by implants and preventing tissue damage associated with rigid components. These properties make hydrogel electrolytes ideal candidates for ZIBs in biomedical applications. In recent years, researchers have not only focused on improving the in vivo safety and biodegradability of hydrogel-based systems, but have also endowed ZIBs with multiple therapeutic functionalities such as antibacterial, anti-inflammatory, and tissue repair capabilities, thereby expanding their potential in biomedical engineering.

**Fig. 11:**
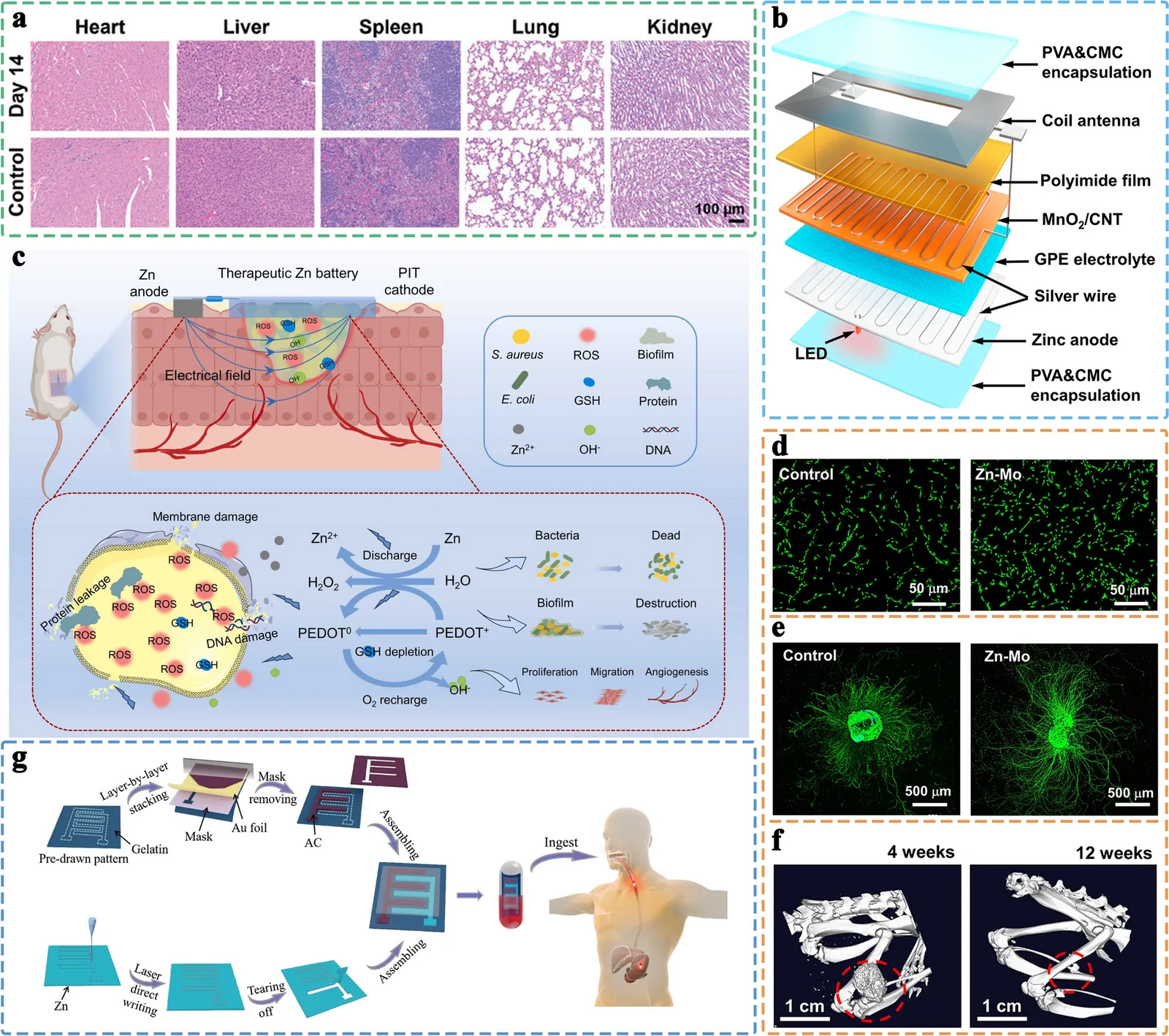
**a** Photographs of major organs collected from the implant group after 14 days and from the control group, showing no apparent inflammation or tissue damage, confirming the excellent biocompatibility of the implantable ZIBs. Reproduced with permission [174]. Copyright 2025, Wiley. **b** Schematic diagram illustrating the design of a wirelessly rechargeable battery system. Reproduced with permission [175]. Copyright 2024, American Chemical Society. **c** Schematic illustration of tailoring electrochemistry of the therapeutic Zn battery for accelerating bacterial-infected chronic wound healing. Reproduced with permission [176]. Copyright 2024, Elsevier. **d** Optical microscopy images showing the growth behavior of Schwann cells after 3 days of culturing in the control and Zn-Mo groups, demonstrating enhanced cell adhesion and proliferation on the Zn-Mo surface. **e** Confocal fluorescence images of DRG after 7 days of culturing, revealing improved neural network formation and excellent cytocompatibility. **f** CT images of rats over 12 weeks following implantation of the Zn-Mo ZIBs, showing no inflammation or tissue damage, confirming long-term biostability and safety. Reproduced with permission [177]. Copyright 2023, American Chemical Society. **g** Schematic illustration of the fabrication process of an edible ZIB. Reproduced with permission [178]. Copyright 2022, American Chemical Society

From a fundamental safety standpoint, traditional implantable batteries may cause inflammation, immune rejection, or tissue damage due to liquid leakage, toxic materials, or rigid structures. Wu et al. designed a silk nanofiber containing zwitterionic hydrogel electrolyte (SPZHE) with excellent electrochemical performance and biocompatibility [174]. In both in vitro and in vivo tests, the material showed no significant toxicity or inflammatory response, while delivering an ionic conductivity of 42.1 mS cm^–1^, a reversible capacity of 524 mF cm^–2^ with 65.1 mWh cm^–2^ energy density, and stable cycling with 80.2% capacitance retention after 2,000 implantation cycles, demonstrating promising biological stability (Fig. 11a).

For implantable medical devices such as cardiac pacemakers and neural stimulators, traditional batteries suffer from limited service life and the need for periodic surgical replacement, which not only increases patient risk and medical burden but also restricts the long-term operation of the device. Wang et al. designed an implantable ZIB using a gelatin/polycaprolactone (PCL) composite hydrogel electrolyte integrated with a wireless charging system, enabling internal energy replenishment without removal (Fig. 11b) [175]. In addition to excellent biodegradability and anti-inflammatory properties, this implantable ZIB also demonstrated a bone-regenerative effect in animal studies; the device delivered a specific capacity of 244.0 mAh g^–1^ (0.5C) with 95.4% retention after 300 cycles and an energy density of 327.1 Wh kg^–1^ and was completely degraded in vivo within 8 weeks, suggesting its potential as a functional material for inflammation suppression and bone repair.

Furthermore, for devices adhered to the skin or implanted near wound sites, the electrochemical process of ZIBs itself can be utilized to regulate the biological microenvironment. Jia et al. developed a wearable ZIB with therapeutic functionality using a dual-conductive hydrogel electrolyte composed of PEDOT and a polyacrylamide–imidazole salt network [176]. The ZIB regulates oxygen species (ROS) and glutathione (GSH) levels to intervene in bacterial infections and inflammation. The therapeutic effects are achieved through a combination of mechanisms: disruption of bacterial membranes via the cationic hydrogel, intracellular ROS burst during discharge, and depletion of GSH in the surrounding microenvironment to sustain oxidative stress. The microcurrent generated enhances endogenous oxidative levels within bacteria, leading to membrane damage and intracellular disruption, ultimately achieving both antibacterial efficacy and biofilm elimination (Fig. 11c).

In addition to infection control and wound healing, flexible ZIBs can also play a significant role in neural tissue engineering. Yin et al. employed a gelatin-based hydrogel electrolyte and utilized the stable electric field generated by a Zn-Mo battery to induce Schwann cell proliferation and dorsal root ganglion (DRG) axon growth, thereby effectively facilitating peripheral nerve regeneration [177]. The ZIB exhibited a marked promotive effect on Schwann cell proliferation and DRG axonal elongation compared to the control group (Fig. 11d, e). This illustrates the battery’s potential to act directly as an electroceutical platform, supporting nerve regeneration. Moreover, the implanted ZIB demonstrated nearly complete biodegradation within 12 weeks in vivo without triggering any significant inflammatory response (Fig. 11f).

In further application explorations, the concept of edible ZIBs has opened new avenues for short-term in vivo diagnostics and gastrointestinal electronic devices. Zhao et al. designed an edible zinc-ion micro-supercapacitor (e-ZMSC) composed of an edible activated carbon microcathode, a zinc microanode, and a gelatin-based hydrogel electrolyte [178]. The device exhibits excellent flexibility, lightweight, and swallowability (Fig. 11g), delivering an energy density of 215.1 μWh cm^–2^ with an areal capacitance of 605 mF cm^–2^ at 1.8 V and retaining 68% capacity after 500 cycles while weighing only ~ 54 mg. The e-ZMSC is capable of powering miniature electronics such as LED lights and gradually degrades in vivo while releasing trace amounts of nutritional zinc, offering both energy supply and functional supplementation. In vivo experiments conducted in a porcine stomach confirmed its feasibility and biosafety under complex physiological conditions.

In summary, the exploration of hydrogel electrolytes in flexible and biocompatible electronic devices has been continuously advancing. These materials not only fulfill fundamental requirements such as flexibility, stability, biocompatibility, and safety, but also endow devices with a range of additional functionalities, such as intelligent sensing, tissue regeneration, and inflammation modulation, through structural optimization and material design. With ongoing improvements in the performance and functionality of hydrogel electrolyte-based ZIBs, their application prospects are expected to expand further in fields including wearable electronics, environmental monitoring, healthcare, and implantable medical devices.

## Summary and Outlook

In this review, we systematically summarized the fundamental characteristics and key design requirements of hydrogel electrolytes in the context of flexible energy storage devices. We clarified the comprehensive performance indicators that these materials must fulfill, including electrochemical properties, mechanical flexibility, safety, and environmental adaptability. On this basis, we discussed diverse compositional design strategies for hydrogel electrolytes, spanning polymer backbones, zinc salts, and functional additives and highlighted advanced functionalization approaches tailored to real-world application scenarios. These include anti-freezing, heat resistance, thermal responsiveness, self-healing, biocompatibility, and sustainability. Such strategies not only enhance the operational safety and environmental resilience of hydrogel electrolytes but also expand their potential in wearable, implantable, and green energy storage systems. Benefiting from structural tunability and multifunctional integration, hydrogel electrolytes are gradually emerging as strong candidates to replace traditional liquid electrolytes. Nevertheless, their widespread practical application still faces several critical challenges. To accelerate the deployment of hydrogel-based energy storage technologies, particularly in flexible and wearable electronics, further breakthroughs are needed in the following key areas:

1. Enhancing Energy Density: Energy density remains a core metric for evaluating energy storage systems, directly determining their endurance and volumetric efficiency. However, the relatively low intrinsic ionic conductivity and bulk thickness of conventional hydrogel electrolytes often lead to increased mass loading and diminished energy density at the device level. To address this bottleneck, future work should focus on the development of ultrathin, low-mass-density hydrogel electrolytes that maintain sufficient ionic conductivity and electrochemical stability.
2. Balancing Mechanical Strength and Flexibility: While ultrathin hydrogel designs offer weight and thickness advantages, they often compromise mechanical robustness, interfacial contact quality, and long-term structural integrity. Thus, future strategies should aim to balance ionic conductivity with mechanical strength. This could involve the incorporation of high-modulus frameworks, construction of hierarchical composite structures, or the design of molecular networks capable of stress dissipation. Additionally, long-term reliability under dynamic deformation, extreme temperature, and humid environments should be prioritized.
3. Improving Interfacial Compatibility and Charge Transfer: The efficiency of interfacial charge transfer critically influences power output. The intimate and stable contact between the hydrogel electrolyte and electrodes is essential for promoting ion transport and minimizing interfacial resistance [102]. However, repeated electrochemical cycling causes volume fluctuations and mechanical stress, leading to delamination and increased resistance. Recent studies have focused on interfacial engineering strategies to alleviate these problems by strengthening adhesion and regulating ion transport. As summarized in Table X, typical approaches, including chemical grafting, SEI regulation, inorganic fillers, zwitterionic modification, and buffer interlayer design, have been demonstrated to enhance interfacial stability and improve charge-transfer kinetics. To address these challenges, considerable efforts have been devoted to interfacial engineering, aiming to reinforce adhesion, homogenize Zn^2+^ flux, and stabilize interfacial chemistry. Strategies such as chemical grafting, SEI regulation, inorganic filler incorporation, zwitterionic modification, and buffer interlayer design have demonstrated remarkable progress in enhancing interfacial compatibility and long-term cycling stability (Table 6). These approaches collectively emphasize that both chemical interaction and mechanical adaptability play indispensable roles in maintaining durable and reversible Zn plating/stripping. Despite these advances, the fundamental understanding of polymer–electrolyte interactions, particularly their influence on the electrical double layer and ion transport mechanisms, remains limited. Further elucidation of these mechanisms is essential for guiding the rational design of next-generation interfacial architectures.
4. Toward Multifunctional and Integrated Design: With the increasing complexity of application scenarios, single-function hydrogel electrolytes are no longer sufficient. The next generation of materials must integrate multiple functions (e.g., self-healing, anti-freezing, thermal resistance) without compromising baseline electrochemical performance. Challenges include maintaining ionic conductivity in the presence of organic solvents, ensuring mechanical strength while achieving rapid self-healing, and expanding the range of thermoresponsive polymer candidates beyond the currently dominant PNIPAM systems.
5. Enhancing Biocompatibility and Sustainability: For applications in wearable medical devices, electronic skin, and implantable electronics, biocompatibility and environmental safety are crucial. Hydrogel electrolytes must avoid causing skin irritation, toxicity, or immune responses. This requires the adoption of non-toxic, biodegradable polymers (e.g., gelatin, chitosan, alginate) and environmentally friendly synthesis routes. In addition, the recyclability, renewability, and biodegradability of materials should be considered throughout the design process to minimize environmental impact.
6. Integrating Artificial Intelligence for Accelerated Design: Artificial intelligence (AI) offers a transformative tool for accelerating the discovery and optimization of hydrogel electrolytes. By combining experimental data and computational simulations into predictive models, AI can identify promising material candidates, guide formulation development, and elucidate complex structure–property relationships [179]. Moreover, AI-assisted theoretical methods, complementing traditional techniques such as density functional theory and molecular dynamics, can significantly reduce the time and cost of performance prediction and mechanistic analysis [180]. The integration of AI into electrolyte research is expected to unlock new pathways for efficient materials screening and high-throughput optimization.

**Table 6:** Summary of representative interfacial engineering strategies for hydrogel electrolytes in ZIBs

Strategy	Mechanism	Example	*R*_ct_ (Ω cm^2^)	Overpotential (mV)	Cycle life	Dendrite suppression	Refs
Buffer interlayer	Stress absorption	PVA-based Zn^2+^ ionic buffer layer	Stable interfacial impedance	Lower overpotential than blank	≥ 45 cycles at 8 mA cm^−2^; blank ≈ 25 cycles (Zn//Zn)	Dendrite-free morphology	[181]
Chemical grafting	Forms covalent bonding	Sulfonate-grafted hydrogel	36.5 (vs. 92.7 for blank)	34 (vs. 110 for blank)	5000 h (Zn//Zn, 1 mA cm^−2^)	Dendrite-free morphology	[182]
SEI	Zn^2+^ flux control	Starch/PAAm-based SEI-forming hydrogel	Smaller R_ct_ vs. blank	–	3200 h (Zn//Zn, 2 mA cm^−2^)	Compact and uniform SEI (~ 30 μm), Dendrite-free morphology	[183]
Inorganic fillers	Electric field redistribution	Boron nitride nanosheets	76 (vs. 169 for blank)	Lower overpotential than blank	1500 h (Zn//Zn, 2 mA cm^−2^)	Dendrite-free morphology	[184]
Zwitterionic modification	Ion regulation	Betaine hydrogel electrolyte	13.7 (vs. 76 for blank)	81 mV (vs. 120–170 for blank)	2000 h (Zn//Zn, 0.5 mA cm^−2^)	Smooth Zn surface, no dendrites after 50 h cycling	[168]

**Fig. 12:**
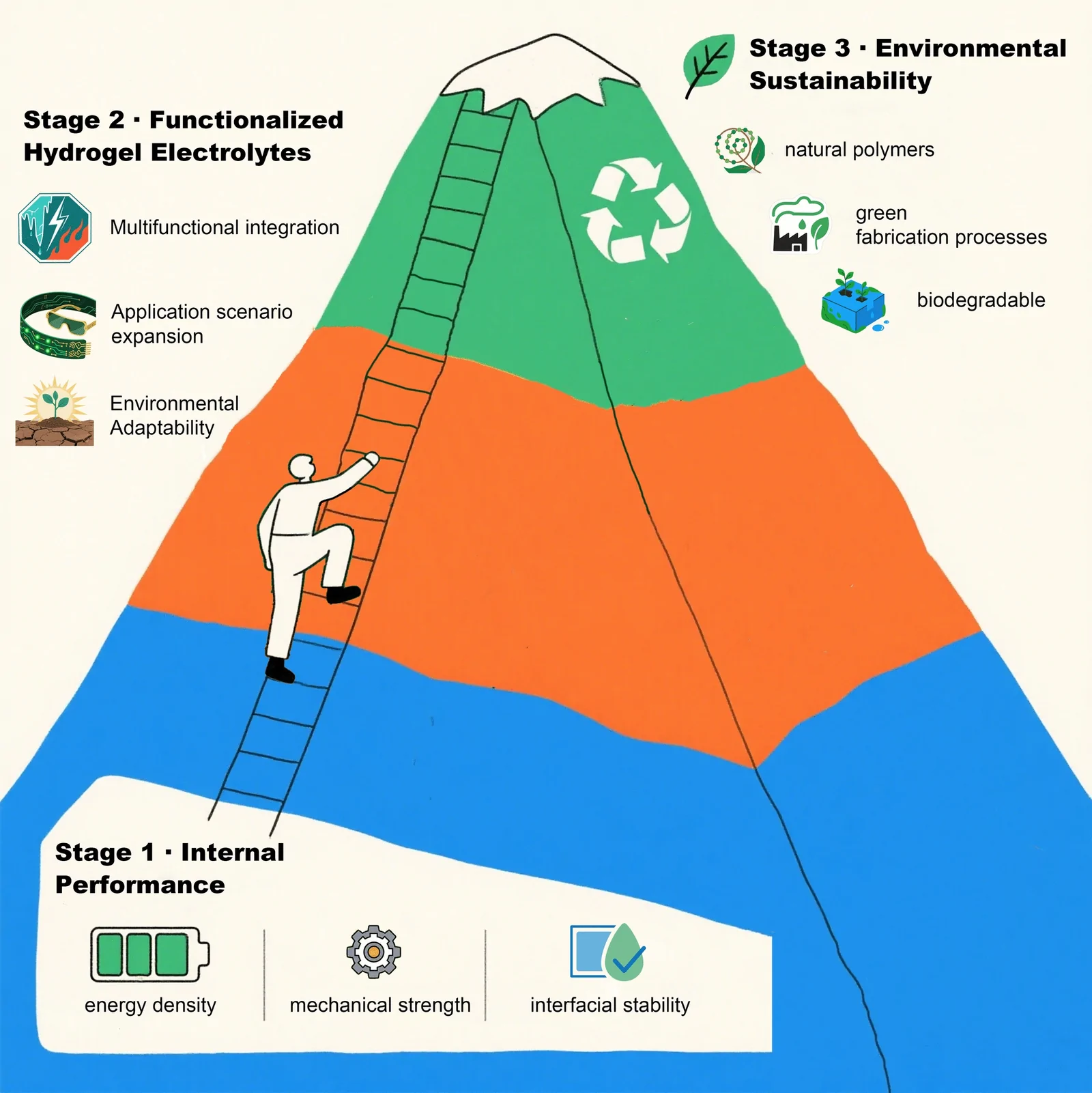
Schematic roadmap for the development of hydrogel electrolytes

As illustrated in Fig. 12, the future development trajectory of hydrogel electrolytes can be broadly divided into three progressive stages. Stage I emphasizes the enhancement of intrinsic material properties, such as energy density, mechanical strength, and interfacial stability, to fulfill the fundamental performance requirements of next-generation energy storage systems. Stage II focuses on the development of multifunctionally integrated hydrogel electrolytes with capabilities such as self-healing, thermal responsiveness, and environmental adaptability. These features are critical for expanding the applicability of hydrogel electrolytes in flexible electronics, wearable devices, and energy storage systems operating under extreme environmental conditions. Stage III centers on environmental sustainability and biocompatibility, advocating the use of natural polymer matrices, green synthesis approaches, and biodegradable designs. This stage ensures the safe, scalable, and eco-friendly deployment of hydrogel electrolytes, thereby enabling the synergistic realization of high performance, multifunctionality, and environmental responsibility in future energy storage technologies.

In summary, the development of next-generation hydrogel electrolytes should move beyond single-parameter optimization toward a comprehensive strategy that simultaneously addresses performance, safety, and sustainability. Through continued innovation in material design, functional integration, and intelligent optimization, hydrogel electrolytes hold great promise for enabling high-performance, durable, and eco-friendly ZIBs for future flexible energy storage technologies.
